# IMGT/HighV-QUEST Statistical Significance of IMGT Clonotype (AA) Diversity per Gene for Standardized Comparisons of Next Generation Sequencing Immunoprofiles of Immunoglobulins and T Cell Receptors

**DOI:** 10.1371/journal.pone.0142353

**Published:** 2015-11-05

**Authors:** Safa Aouinti, Dhafer Malouche, Véronique Giudicelli, Sofia Kossida, Marie-Paule Lefranc

**Affiliations:** 1 IMGT, the international ImMunoGeneTics information system, Laboratoire d’ImmunoGénétique Moléculaire LIGM, Institut de Génétique Humaine IGH, UPR CNRS 1142, Montpellier University, Montpellier cedex 5, France; 2 Higher School of Statistics and Information Analysis, University of Carthage, Tunis, Tunisia; 3 National School of Engineers of Tunis, Information Technology and Communications Department, Laboratory U2S, University of Tunis El-Manar, Tunis, Tunisia; University of London, St George’s, UNITED KINGDOM

## Abstract

The adaptive immune responses of humans and of other jawed vertebrate species (gnasthostomata) are characterized by the B and T cells and their specific antigen receptors, the immunoglobulins (IG) or antibodies and the T cell receptors (TR) (up to 2.10^12^ different IG and TR per individual). IMGT, the international ImMunoGeneTics information system (http://www.imgt.org), was created in 1989 by Marie-Paule Lefranc (Montpellier University and CNRS) to manage the huge and complex diversity of these antigen receptors. IMGT built on IMGT-ONTOLOGY concepts of identification (keywords), description (labels), classification (gene and allele nomenclature) and numerotation (IMGT unique numbering), is at the origin of immunoinformatics, a science at the interface between immunogenetics and bioinformatics. IMGT/HighV-QUEST, the first web portal, and so far the only one, for the next generation sequencing (NGS) analysis of IG and TR, is the paradigm for immune repertoire standardized outputs and immunoprofiles of the adaptive immune responses. It provides the identification of the variable (V), diversity (D) and joining (J) genes and alleles, analysis of the V-(D)-J junction and complementarity determining region 3 (CDR3) and the characterization of the ‘IMGT clonotype (AA)’ (AA for amino acid) diversity and expression. IMGT/HighV-QUEST compares outputs of different batches, up to one million nucleotide sequencesfor the statistical module. These high throughput IG and TR repertoire immunoprofiles are of prime importance in vaccination, cancer, infectious diseases, autoimmunity and lymphoproliferative disorders, however their comparative statistical analysis still remains a challenge. We present a standardized statistical procedure to analyze IMGT/HighV-QUEST outputs for the evaluation of the significance of the IMGT clonotype (AA) diversity differences in proportions, per gene of a given group, between NGS IG and TR repertoire immunoprofiles. The procedure is generic and suitable for evaluating significance of the IMGT clonotype (AA) diversity and expression per gene, and for any IG and TR immunoprofiles of any species.

## Introduction

IMGT, the international ImMunoGeneTics information system (http://www.imgt.org) [1], was created in 1989 by Marie-Paule Lefranc, Laboratoire d’ImmunoGénétique Moléculaire LIGM (Montpellier University and CNRS) at Montpellier, France, in order to standardize and to manage the complexity and the diversity of immunogenetics data. IMGT, built on IMGT-ONTOLOGY [2], is at the origin of immunoinformatics [3], a science at the interface between immunogenetics and bioinformatics.

The adaptive immune response was acquired by jawed vertebrates (or gnathostomata) more than 450 million years ago and is found in all extant jawed vertebrate species from fishes to humans [3]. The potential antigen receptor repertoire of each individual is estimated to comprise about 10^12^ different immunoglobulins (IG) or antibodies [4] and 10^12^ different T cell receptors (TR) [5] per individual. This huge diversity is created by combinatorial and junctional diversity (together with somatic hypermutations for IG) and the limiting factor is only the number of B and T cells that an organism is genetically programmed to produce [3].

IG are made of two identical heavy (H) chains and two identical light (L) (kappa or lambda) chains, encoded by genes located in three major loci: the IG heavy (IGH) locus, IG kappa (IGK) locus and IG lambda (IGL) locus [4, 6]. TR are made of two chains, alpha and beta, or gamma and delta, encoded by genes located in four major loci: the TR alpha (TRA), TR beta (TRB), TR gamma (TRG) and TR delta (TRD) [5, 7] (see IMGT Repertoire, http://www.imgt.org/IMGTindex/locus.html and IMGT/GENE-DB [8]).

There are four IG or TR gene types: variable (V), diversity (D) (only for IGH, TRB and TRD), joining (J) and constant (C) genes, which define 24 IG and TR groups (e.g., IGHV, IGHD, IGHJ, …, TRBV, TRBD, TRBJ, …) [2, 3] (http://www.imgt.org/IMGTindex/group.html). The V, D, J and C genes contribute to the IG and TR chain synthesis [3–5]. The variable domain at the N-terminal end of each IG or TR chain results from a V-(D)-J rearrangement whereas the remaining of the chain, or constant region, is encoded by a C gene [3–5].

The analysis of the immune antigen receptor (IG and TR) repertoires has greatly benefited from the next generation sequencing (NGS) technologies. The vast amount of generated data necessitated the development of novel methods and analysis tools. IMGT/HighV-QUEST [9], a high throughput version of IMGT/V-QUEST [10–14] was implemented by IMGT in October 2010 and is the first reference NGS web portal for IG and TR. IMGT/HighV-QUEST analyzes up to 1,000,000 IG and TR sequences from NGS high throughput and deep sequencing [9, 15, 16] and compares outputs of different batches, up to one million nucleotide sequences for the statistical module. The analysis is based on the IMGT-ONTOLOGY concepts of identification, description, classification and numerotation [2].

IMGT/HighV-QUEST [9, 15] uses the same algorithm as IMGT/V-QUEST [10–14]. It identifies the variable (V), diversity (D) and joining (J) genes and alleles, describes the V-REGION mutations, determines the hot spot positions in the closest germline V gene and allele, incorporates IMGT/JunctionAnalysis for the analysis of the V-J and V-D-J junctions [17, 18] and IMGT/Automat [19, 20] for a full V-J-REGION and V-D-J-REGION annotation.

As a novel NGS functionality, IMGT/HighV-QUEST identifies clonotypes (for IG or TR) which are defined as ‘IMGT clonotype (AA)’ (AA for amino acid) and are characterized by a unique V-(D)-J rearrangement (IMGT genes and alleles determined at the nucleotide level), conserved CDR3-IMGT anchors (cysteine C 104, tryptophan W 118 or phenylalanine F 118), and a unique CDR3-IMGT AA junction sequence [16]. Each ‘IMGT clonotype (AA)’ is characterized by a selected unique representative sequence. For the first time for NGS antigen receptor data analysis, the IMGT standardized approach allows a clear distinction between the clonotype diversity (numbers of IMGT clonotypes (AA) per V, D or J gene), and the clonotype expression (numbers of sequences assigned, unambiguously, to a given IMGT clonotype (AA) per V, D or J gene) [16], making the IMGT/HighV-QUEST NGS immunoprofiles available for statistical analysis.

In this work, we analyze IMGT/HighV-QUEST NGS outputs on IMGT clonotypes (AA) diversity per gene between NGS sets with the aim of defining a standardized IMGT/HighV-QUEST statistical procedure for the evaluation of the significance of the IMGT clonotype (AA) diversity differences in proportions between high throughput repertoire immunoprofiles.

## Materials and Methods

### IMGT/HighV-QUEST outputs

IMGT/HighV-QUEST outputs are from the analysis performed in [16] which aimed to minimize experimental biases. The analysis of T cell receptors results were selected as they do not undergo somatic mutations as opposed to immunoglobulins. The choice of the 454 Genome Sequencer FLX (GSFLX) Titanium (Roche) technology allowed to obtain sequences long enough to cover the full V-DOMAIN [3]. Amplicon libraries of the corresponding TRB V-D-J transcripts were prepared using anchored 5’ rapid amplification of cDNA ends (RACE) [21, 22] polymerase chain reaction (PCR) to exclude amplification bias. The quality of the results was confirmed by the identification of transcripts of pseudogenes (previously not known as being transcribed) and by the strikingly similar pattern of distribution of TRBV genes and alleles of the common IMGT clonotypes (AA) between sets of a given T cell population at different time points [16]. Sequencing data is available in the NCBI Sequence Read Archive under the accession code SRX326382 [16].

Eight from the twelve sets analyzed in [16] were selected for this study. They correspond to two T cell populations (CD4^-^ and CD4^+^) at four time points (pre-vaccination (Pre), and day 3 (d3), day 8 (d8) and day 26 (d26) post-vaccination) of a single individual vaccinated against H1N1 influenza virus. The sets are identified by barcodes or multiplex identifiers (MID) (MID1, MID2, MID4, MID5, MID7, MID8, MID10 and MID11) (Table 1) which allow to differentiate between the two T cell populations and the four time points. The reads were run with IMGT/HighV-QUEST program version 1.1.3, IMGT/V-QUEST program version 3.2.31 and IMGT/V-QUEST reference directory release 201338-1 (http://www.imgt.org) [9, 15, 16].

**Table 1:** Number (nb) of in-frame *Homo sapiens* TRB IMGT clonotypes (AA) per group in CD4^-^ versus (∼) CD4^+^ populations at four time points.

Compared sets* CD4^-^(set *i*) ∼ CD4^+^(set *j*)	Time points	Nb of IMGT clonotypes (AA)** per group in CD4^-^(n_*i*_) ∼ CD4^+^(n_*j*_)		
		TRBV	TRBD	TRBJ
CD4^-^(MID1) ∼ CD4^+^(MID2)	Pre	2234 ∼ 2523	2124 ∼ 2412	2234 ∼ 2523
CD4^-^(MID4) ∼ CD4^+^(MID5)	d3	1389 ∼ 2859	1323 ∼ 2751	1389 ∼ 2859
CD4^-^(MID7) ∼ CD4^+^(MID8)	d8	1309 ∼ 1966	1242 ∼ 1886	1309 ∼ 1966
CD4^-^(MID10) ∼ CD4^+^(MID11)	d26	1924 ∼ 2875	1853 ∼ 2769	1924 ∼ 2875

* Sets identified by barcodes or multiplex identifiers (MID).

** IMGT clonotypes (AA) are defined by a unique V-(D)-J rearrangement and characterized by their V gene and J gene [16] (therefore identical *n*_*i*_ and *n*_*j*_ per TRBV and TRBJ group at a given time point). The lower numbers of IMGT clonotypes (AA) for TRBD group *n*_*i*_ ∼ *n*_*j*_ are due to the difficulty of identifying the TRBD genes and alleles, owing to their short length and high % of identity [5]. Six IMGT clonotypes (AA) with an outlier CDR3-IMGT length (> 60 AA or < 4 AA) were removed: two from MID1 (63 AA and 65 AA), two from MID2 and MID5 (2 AA) and two from MID2 and MID10 (3 AA).

### IMGT/HighV-QUEST statistical procedure

#### Differences in proportions

The purpose was to compare, between two sets, the differences in proportions of IMGT clonotypes (AA) per gene of a given group (clonotype diversity). For this analysis, only IMGT clonotypes (AA) which are in-frame (no frameshift in the junction) identified by IMGT/HighV-QUEST are selected. Statistical analyses applied to data sets were performed using R software (www.r-project.org; version 3.2.0).

Let *m* be the number of genes analyzed in a given group of a locus, for example, the *Homo sapiens* TRB locus [8], 54 genes for the TRBV group, 2 genes for the TRBD group and 13 genes for the TRBJ group. Let each gene of a given group, identified by its IMGT gene name (e.g., TRBV2, TRBJ1-1…) [5, 7] be indexed by *k* (*k* = 1, …, *m*).

Sets to be compared corresponding to T cell populations CD4^-^ and CD4^+^ were indexed by {*i*, *j*} (*i* ≠ *j*). The numbers (nb) of IMGT clonotypes (AA) per group (TRBV, TRBD or TRBJ) in CD4^-^ versus CD4^+^ populations (*n*_*i*_ ∼ *n*_*j*_) at four time points (Pre, d3, d8 and d26) are shown in Table 1.

Let ${\left(X_{r}\right)}_{r=1..n_{i}}^{k}$ and ${\left(Y_{s}\right)}_{s=1..n_{j}}^{k}$ be two random variables defined as follows:

$${\left(X_{r}\right)}_{r=1..n_{i}}^{k}=\left\{\begin{array}{c} 1\text{,}\;\text{if}\;\text{the}\;r^{th}\;\text{IMGT}\;\text{clonotype}\;\text{(AA)}\;\text{in}\;\text{the}\;\text{set}\;i\\ \;\;\text{with}\;\text{the}\;\text{gene}\;k\;\text{of}\;\text{a}\;\text{given}\;\text{group}\\ 0\text{,}\;\text{else} \end{array}\right.$$

and

$${\left(Y_{s}\right)}_{s=1..n_{j}}^{k}=\left\{\begin{array}{c} 1\text{,}\;\text{if}\;\text{the}\;s^{th}\;\text{IMGT}\;\text{clonotype}\;\text{(AA)}\;\text{in}\;\text{the}\;\text{set}\;j\\ \;\;\text{with}\;\text{the}\;\text{gene}\;k\;\text{of}\;\text{a}\;\text{given}\;\text{group}\\ 0\text{,}\;\text{else} \end{array}\right.$$

where *n*_*i*_ (resp., *n*_*j*_) is the number of IMGT clonotypes (AA) in the set *i* (resp., set *j*) for a given group (Table 1).

${\left(X_{r}\right)}_{r=1..n_{i}}^{k}$ (resp., ${\left(Y_{s}\right)}_{s=1..n_{j}}^{k}$) were considered to be independent and identically distributed (i.i.d.) Bernoulli random variables of parameter $p_{i}^{k}$ (resp., $p_{j}^{k}$). The parameters $p_{i}^{k}$ and $p_{j}^{k}$ vary in Θ = [0, 1]^2^ and represent the probabilities of finding at least one IMGT clonotype (AA) with the gene *k* in the set *i* and the set *j*, respectively. ${\hat{p}}_{i}^{k}$ and ${\hat{p}}_{j}^{k}$ are the estimators of $p_{i}^{k}$ and $p_{j}^{k}$, respectively, defined as follows:

$$\left\{\begin{array}{c} {\hat{p}}_{i}^{k}=\frac{\text{Number}\;\text{of}\;\text{IMGT}\;\text{clonotypes}\;\text{(AA)}\;\text{in}\;\text{the}\;\text{set}\;i\;\text{with}\;\text{the}\;\text{gene}\;k\;\text{of}\;\text{a}\;\text{given}\;\text{group}}{\text{Number}\;\text{of}\;\text{IMGT}\;\text{clonotypes}\;\text{(AA)}\;\text{in}\;\text{the}\;\text{set}\;i\;\text{for}\;\text{a}\;\text{given}\;\text{group}}\\ \;=\frac{1}{n_{i}}\sum_{r=1}^{n_{i}}X_{r}\\ {\hat{p}}_{j}^{k}=\frac{\text{Number}\;\text{of}\;\text{IMGT}\;\text{clonotypes}\;\text{(AA)}\;\text{in}\;\text{the}\;\text{set}\;j\;\text{with}\;\text{the}\;\text{gene}\;k\;\text{of}\;\text{a}\;\text{given}\;\text{group}}{\text{Number}\;\text{of}\;\text{IMGT}\;\text{clonotypes}\;\text{(AA)}\;\text{in}\;\text{the}\;\text{set}\;j\;\text{for}\;\text{a}\;\text{given}\;\text{group}}\\ \;=\frac{1}{n_{j}}\sum_{s=1}^{n_{j}}Y_{s} \end{array}\right.$$

For example, the number of IMGT clonotypes (AA) with the gene *k* TRBV5-1 of the TRBV group is 244 in the set i MID1 and 420 in the set j MID2 (S1 Table). Knowing that the number of IMGT clonotypes (AA) is 2234 in the set i MID1 and 2523 in the set j MID2 (Table 1), the estimators of $p_{\text{MID1}}^{\text{TRBV5-1}}$ and $p_{\text{MID2}}^{\text{TRBV5-1}}$ are as follows:

$$\left\{\begin{array}{c} {\hat{p}}_{\text{MID1}}^{\text{TRBV5-1}}=\frac{244}{2234}=0.109221\\ {\hat{p}}_{\text{MID2}}^{\text{TRBV5-1}}=\frac{420}{2523}=0.166468 \end{array}\right.$$

The difference in proportions ${\hat{p}}_{i}^{k}-{\hat{p}}_{j}^{k}$ for TRBV5-1 between MID1 and MID2 is -0.057247 (S1 Table).

Approximate 95% confidence intervals (CI) [23, 24] were calculated for reference to provide a magnitude of the true difference in proportions $p_{i}^{k}-p_{j}^{k}$ as follows:

$$\begin{array}{c} \left({\hat{p}}_{i}^{k}-{\hat{p}}_{j}^{k}\right)\pm z_{\left(1-\frac{\alpha }{2}\right)}.\sigma_{{\hat{p}}_{i}^{k}-{\hat{p}}_{j}^{k}} \end{array}$$ (1)

where $z_{\left(1-\frac{\alpha }{2}\right)}$ is the $1-\frac{\alpha }{2}$ percentile of a standard normal distribution (assumption of the distribution proven in Step 2) (so, at *α* = 5%, $1-\frac{\alpha }{2}=0.975$ and *z* = 1.96) and where $\sigma_{{\hat{p}}_{i}^{k}-{\hat{p}}_{j}^{k}}$ is the standard deviation (SD) (square root of the variance) estimated as follows:

$$\begin{array}{c} \sigma_{{\hat{p}}_{i}^{k}-{\hat{p}}_{j}^{k}}=\sqrt{\frac{{\hat{p}}_{i}^{k}\left(1-{\hat{p}}_{i}^{k}\right)}{n_{i}}+\frac{{\hat{p}}_{j}^{k}\left(1-{\hat{p}}_{j}^{k}\right)}{n_{j}}} \end{array}$$ (2)

For the example of the TRBV5-1 gene, the standard deviation is calculated as follows: $\sigma_{{\hat{p}}_{\text{MID1}}^{\text{TRBV5-1}}-{\hat{p}}_{\text{MID2}}^{\text{TRBV5-1}}}=\sqrt{\frac{0.109221(1-0.109221)}{2234}+\frac{0.166468(1-0.166468)}{2523}}=0.009927$ and the 95% CI for the difference in proportions ${\hat{p}}_{\text{MID1}}^{\text{TRBV5-1}}-{\hat{p}}_{\text{MID2}}^{\text{TRBV5-1}}$ is given by: -0.057247±1.96(0.009927) = [-0.076704, -0.037790] (S1 Table).

#### Significance of the differences in proportions

The hypothesis to be tested is to determine whether the difference between two proportions is significant. In order to evaluate the significance of the differences in proportions, a two-tailed statistical test was performed by following these steps:

**Step 1.** Set up the null hypothesis $H_{0}^{k}$ (there is no difference between $p_{i}^{k}$ and $p_{j}^{k}$) and its alternative $H_{1}^{k}$ defined as follows:

$$\begin{array}{c} \left\{\begin{array}{c} H_{0}^{k}:p_{i}^{k}-p_{j}^{k}=0\\ H_{1}^{k}:p_{i}^{k}-p_{j}^{k}\neq 0 \end{array}\right. \end{array}$$

**Step 2.** In this study, the sample size is large enough to expect a standard normal distribution and to choose the *z*-score test statistic ($z=\frac{\text{score}-\mu (\text{mean})}{\sigma (\text{standard}\;\text{deviation}\;\text{SD})})$ which defines the number of standard deviations an element (here, the difference in proportions ${\hat{p}}_{i}^{k}-{\hat{p}}_{j}^{k}$) is away from the mean (here, the hypothesized difference in proportions $p_{i}^{k}-p_{j}^{k}$), with

$$\begin{array}{c} z^{k}=\frac{\left({\hat{p}}_{i}^{k}-{\hat{p}}_{j}^{k}\right)-\mu_{{\hat{p}}_{i}^{k}-{\hat{p}}_{j}^{k}}}{\sigma_{{\hat{p}}_{i}^{k}-{\hat{p}}_{j}^{k}}}=\frac{\left({\hat{p}}_{i}^{k}-{\hat{p}}_{j}^{k}\right)-\left(p_{i}^{k}-p_{j}^{k}\right)}{\sigma_{{\hat{p}}_{i}^{k}-{\hat{p}}_{j}^{k}}} \end{array}$$ (3)

where $p_{i}^{k}-p_{j}^{k}$ replaces $\mu_{{\hat{p}}_{i}^{k}-{\hat{p}}_{j}^{k}}$ in the classical *z*-score formula.

To reject or accept the null hypothesis a significance level of 5% (*α* = 0.05) was chosen. This is a two-tailed test since a value on either side of the standard normal distribution (*z* > 1.96 or *z* < 1.96) would cause one to reject the null hypothesis.

Note that in the case of null or too small proportions (where $n_{i}{\hat{p}}_{i}^{k}<5,n_{i}\left(1-{\hat{p}}_{i}^{k}\right)<5$ and $n_{j}{\hat{p}}_{j}^{k}<5,n_{j}\left(1-{\hat{p}}_{j}^{k}\right)<5$), the *z*-score results are not considered (e.g., the case of the TRBV5-4 and TRBV7-7 genes having a null proportion in the set MID1).

Under the null hypothesis $H_{0}^{k}$, the difference $p_{i}^{k}-p_{j}^{k}$ is null and the z-score formula (3) becomes as follows:

$$z^{k}=\frac{{\hat{p}}_{i}^{k}-{\hat{p}}_{j}^{k}}{\sigma_{{\hat{p}}_{i}^{k}-{\hat{p}}_{j}^{k}}}∼N\left(0,1\right)$$ (4)

Replacing in Eq (4) the denominator $\sigma_{{\hat{p}}_{i}^{k}-{\hat{p}}_{j}^{k}}$ Eq (2) by the pooled estimate of the standard deviation $\sigma_{{\hat{p}}_{ij}^{k}}$ of the weighted pooled proportion ${\hat{p}}_{ij}^{k}=\frac{n_{i}{\hat{p}}_{i}^{k}+n_{j}{\hat{p}}_{j}^{k}}{n_{i}+n_{j}}$[25, 26], the *z*-score formula becomes as follows:

$$\begin{array}{c} z^{k}=\frac{{\hat{p}}_{i}^{k}-{\hat{p}}_{j}^{k}}{\sqrt{{\hat{p}}_{ij}^{k}\left(1-{\hat{p}}_{ij}^{k}\right)\left(\frac{1}{n_{i}}+\frac{1}{n_{j}}\right)}}∼N\left(0,1\right) \end{array}$$ (5)

The pooled estimation of the standard deviation is valid only if the null hypothesis is true and it is used in order to reduce the variability of the difference between the two tested sample proportions. Thus, the weighted pooled proportion allows the control of this variability by assigning more weight to the larger sample size.

For the example of TRBV5-1 gene, if the null hypothesis $H_{0}^{k}:p_{\text{MID1}}^{\text{TRBV5-1}}=p_{\text{MID2}}^{\text{TRBV5-1}}$ is true, the weighted pooled proportion ${\hat{p}}_{ij}^{k}$ is ${\hat{p}}_{\text{MID1MID2}}^{\text{TRBV5-1}}=\frac{\left(2234\times 0.109221)+(2523\times 0.166468\right)}{2234+2523}=0.139583$, the pooled estimate of the standard deviation $\sigma_{{\hat{p}}_{ij}^{k}}$ is $\sigma_{{\hat{p}}_{\text{MID1MID2}}^{\text{TRBV5-1}}}=\sqrt{0.139583\left(1-0.139583\right)\left(\frac{1}{2234}+\frac{1}{2523}\right)}=0.010067$ and the test statistic is $z^{\text{TRBV5-1}}=\frac{-0.057247}{0.010067}=-5.686$ (S2 Table).

At the significance level of 5% (*α* = 0.05) level and two-tailed test, the null hypothesis $H_{0}^{\text{TRBV5-1}}$ is rejected since -5.686 (more than 5 standard deviations from zero) is much less than -1.96 and falls in the $\frac{\alpha }{2}=0.025$ left zone of the standard normal distribution.

**Step 3.** Each *z*-score for each difference in proportions leads to a *p*-value obtained from computed tables. The *p*-value is the probability P of observing a value Z greater than the absolute value of the *z*-score observed under the null hypothesis *H*_0_. The *p*-value allows, as the critical value (step 2), to reject or not the null hypothesis, at a defined rejection level (usually *α* = 0.01 or 0.05 or 0.1) (the critical value approach (step 2) and the *p*-value approach should always lead to the same conclusion). It also allows to evaluate the significance of the deviation from the null hypothesis. The *p*-value calculation is based on the alternative hypothesis $H_{1}^{k}$ as follows: 2P(Z≥∣z∣) for $H_{1}^{k}:p_{i}^{k}\neq p_{j}^{k}$ (i.e., P(Z≥z) for $H_{1}^{k}:p_{i}^{k}>p_{j}^{k}$ and P(Z≤z) for $H_{1}^{k}:p_{i}^{k}<p_{j}^{k}$).

For the example of the TRBV5-1 gene, the computed *p*-value 2P(Z≥∣5.686∣) is 1.3E-08. Since this *p*-value is much less than the rejection level 0.05, the null hypothesis is rejected and the difference in proportions of the TRBV5-1 gene between MID1 and MID2 is declared to be significant at *α* = 0.05.

#### Error rates control and multiple testing procedures

The intuitive idea is to test *p*-value obtained for each gene, individually from each *z*-score test at a chosen *α* level (usually *α* = 0.05, implying that it is acceptable to have a 5% probability of incorrectly rejecting the null hypothesis). However, when multiple tests of hypotheses are conducted simultaneously, more than 5% of them are very likely to be statistically significant purely by chance; for example, when *m* tests (here, number of genes) are performed, the experimentwise significance level will be given by 1 − (1 − *α*)^*m*^ ≈ *mα* when *α* is small and will represent the global Type I error rate (i.e., risk to incorrectly reject a true null hypothesis *H*_0_ (false positive), here, for instance, the difference in proportions of IMGT clonotypes (AA) with the gene *k* between two sets would be declared as significant while this is not the case). For that reason, an adjustment of the *p*-values must be made through a multiple testing procedure.

There are two basic and commonly used strategies for dealing with multiple hypotheses testing by controlling different error rates which incorporate the number of performed tests: the family-wise error rate (FWER) defined by Tukey [27] and the false discovery rate (FDR) of Benjamini & Hochberg [28]. The FWER is the probability to make one or more Type I errors or false positives (i.e., when the statistical test leads to reject a null hypothesis $H_{0}^{k}$ when it is true) among all the *m* hypotheses tested, whereas the FDR is the expected proportion of Type I errors among rejected hypotheses. The FDR procedures are generally more powerful (i.e., they provide a high probability that the null hypothesis $H_{0}^{k}$ is correctly rejected by the statistical test when the alternative hypothesis $H_{1}^{k}$ is true) than the FWER procedures, considered as more conservative [28–30]. Therefore, the FDR procedures are generally applied in an explanatory context or in the case of large scale multiple testing problems. The FWER procedures are more appropriate in confirmatory or regulatory setting due to their strict control of risk of Type I errors. In some situations, the FWER is likely to be a secure measure of controlling the error rates especially when the test is falsely declared as significant. For these reasons, the choice of the adequate error measure is heavily influenced by the scientific question asked.

In this study, the following multiple testing procedures were applied: Bonferroni [31], Holm [32], Hochberg [33], ŠidákSS and ŠidákSD [34, 35], controlling the FWER, as well as Benjamini & Hochberg (BH) [28] and Benjamini & Yekutieli (BY) [36], controlling the FDR (Table 2). Each procedure belongs to one of the two main categories of algorithms for generating multiple testing adjustments: single-step and stepwise procedures (step-down or step-up algorithms). In single-step procedures, a single criterion is used to evaluate the importance of all test statistics or corresponding *p*-values without taking into consideration their order or their number, namely, a critical value is used to assess each null hypothesis $H_{0}^{k}$ independently of the results obtained by testing all the other hypotheses [37].

**Table 2:** Properties of the multiple testing procedures.

**Procedures**	**Type of control**	**Algorithm structure**	**Dependence of p-values under *H*_0_**	**Properties**
**Bonferonni** [31]	FWER	*Single-step*	Ignorance	The most conservative
**Šidák (SS)** [34, 35]	FWER	*Single-step*	Independence	Less conservative than Bonferroni
**Holm** [32]	FWER	*Step-down*	Ignorance	Less conservative than Bonferroni
**Šidák (SD)** [34, 35]	FWER	*Step-down*	Dependence	Similar to Holm
**Hochberg** [33]	FWER	*Step-up*	Independence	*Step-up* of Holm
**Benjamini & Hochberg (BH)** [28]	FDR	*Step-up*	Independence	The least conservative
**Benjamini & Yekutieli (BY)** [36]	FDR	*Step-up*	Ignorance	More conservative than BH

Stepwise procedures take into account results obtained for the previous hypotheses. They are improved in terms of power while maintaining the control of the Type I error rate. Tests are considered successively either in descending order of their corresponding *p*-values (step-down) or in ascending order (step-up). For step-down procedures, null hypotheses having the largest test statistic (smallest unadjusted *p*-values) are regarded successively. As soon as one of the null hypotheses does not get rejected, the step-down procedure stops and no more hypotheses are rejected. For step-up procedures, null hypotheses having the least test statistic (largest unadjusted *p*-values) are regarded successively. As soon as one of the null hypotheses is rejected, the step-up procedure stops and all residual more significant hypotheses are rejected [34].

All procedures mentioned above take into account the total number *m* of tested hypotheses. For this reason and in order to refine the results by reducing the error risks, TRBV genes not found in the IMGT clonotypes (AA) of the two compared sets (i.e., the difference in proportions is null) were excluded when applying multiple testing procedures.

## Results

### IMGT clonotypes (AA) for 10,000 per group

**Fig 1:**
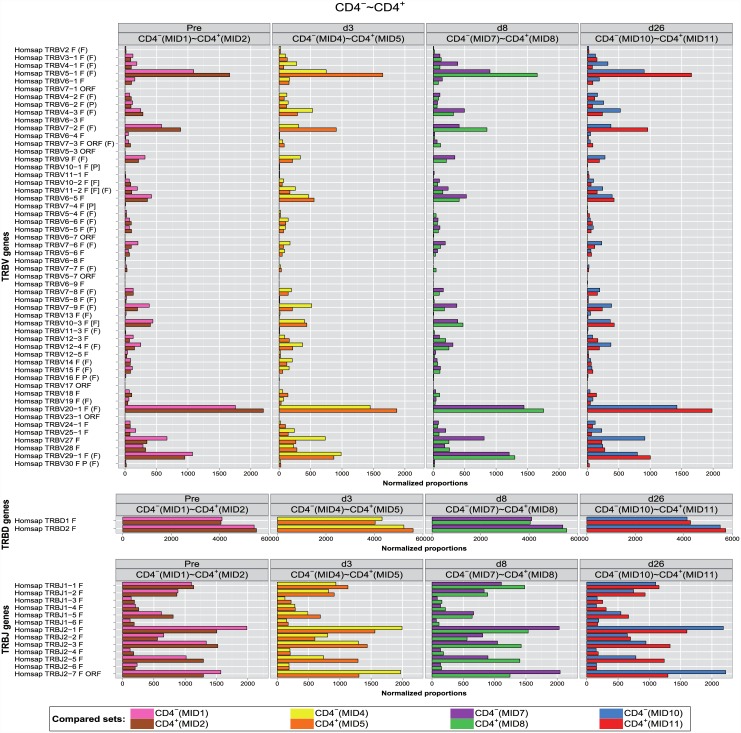
Normalized bar graphs of the proportions. The normalized bar graphs of the proportions for *Homo sapiens* TRB IMGT clonotypes (AA) with a gene of a given group (TRBV, TRBD or TRBJ) are shown between two T cell populations (CD4^-^ and CD4^+^) at four time points (Pre, d3, d8 and d26). IMGT clonotypes (AA) proportions are normalized for 10,000 IMGT clonotypes (AA) per group. Juxtaposed colored bars in each panel correspond to CD4^-^ (top) and CD4^+^ (bottom).

As a first step, in order to permit a visual comparative analysis of IMGT clonotype (AA) diversity for each gene per group (TRBV, TRBD or TRBJ) between two sets, the numbers of IMGT clonotypes (AA) for a given gene obtained from the IMGT/HighV-QUEST outputs were normalized for 10,000 IMGT clonotypes (AA) per group and represented by bar graphs (Fig 1) for the CD4^-^ and CD4^+^ populations (juxtaposed colored top and bottom bars, respectively). The four panels correspond to the four time points (Pre, d3, d8, d26).

Differences in proportions and corresponding 95% CI were calculated for the IMGT clonotypes (AA) with a gene *k* for a given group (TRBV, TRBD or TRBJ), between the two T cell populations (CD4^-^ and CD4^+^) at four time points (Pre, d3, d8, d26). S1 Table reports, as examples, the values for the differences in proportions found significant in this study (discussed in next paragraphs).

### Significance using mutiple testing procedures

In order to evaluate the significance of the differences in proportions found in the two T cell populations CD4^-^ and CD4^+^, at the four time points, the multiple testing procedures cited in Material & Methods (Table 2) were applied. Unadjusted *p*-values and adjusted *p*-values from the multiple testing procedures were calculated. In Table 3 are shown the smallest unadjusted *p*-values (rawp) (< 0.05) and the corresponding adjusted values after application of the FWER (Bonferroni, Holm, Hochberg, ŠidákSS and ŠidákSD) or FDR (BH and BY) controlling procedures. Adjusted *p*-values below the rejection level 0.05 under a given procedure are highlighted in bold and correspond to significant tests (Table 3).

**Table 3:** Unadjusted and adjusted *p*-values from multiple testing procedures. Unadjusted *p*-values (rawp) and adjusted *p*-values from multiple testing procedures were calculated, for the significance of the differences in proportions for *Homo sapiens* TRB IMGT clonotypes (AA) with a gene of a given group (TRBV, TRBD or TRBJ), between two T cell populations (CD4^-^ and CD4^+^) at four time points (Pre, d3, d8 and d26).

**Compared sets [time points]**	**Unadjusted *p*-values (rawp)**	**Adjusted p-values from multiple testing procedures**						
		**Bonferroni**	**Holm**	**Hochberg**	**ŠidákSS**	**ŠidákSD**	**BH**	**BY**
**CD4^-^(MID1) ∼ CD4^+^(MID2) [Pre]**								
Homsap TRBV5-1 F (F)	1.3E-08	**7.93E-07**	**7.93E-07**	**7.93E-07**	**7.93E-07**	**7.93E-07**	**7.93E-07**	**3.72E-06**
Homsap TRBV27 F	4.81E-07	**2.93E-05**	**2.89E-05**	**2.89E-05**	**2.93E-05**	**2.89E-05**	**1.47E-05**	**6.89E-05**
Homsap TRBJ2-1 F	1.2E-05	**0.00073**	**0.00071**	**0.00071**	**0.00073**	**0.00071**	**0.00024**	**0.00114**
Homsap TRBV7-2 F (F)	7E-05	**0.00447**	**0.00425**	**0.00425**	**0.00446**	**0.00424**	**0.00112**	**0.00525**
Homsap TRBV7-9 F (F)	0.00011	**0.00697**	**0.00652**	**0.00652**	**0.00695**	**0.00649**	**0.00127**	**0.00596**
Homsap TRBV20-1 F (F)	0.00012	**0.00761**	**0.00699**	**0.00699**	**0.00758**	**0.00696**	**0.00127**	**0.00596**
Homsap TRBV7-6 F (F)	0.00243	0.14798	0.13343	0.13343	0.13771	0.12505	**0.02114**	0.09928
Homsap TRBJ2-5 F	0.00304	0.18524	0.16398	0.16398	0.16933	0.15145	**0.02315**	0.10874
Homsap TRBV25-1 F	0.00439	0.26766	0.23256	0.23256	0.23528	0.2079	**0.02974**	0.13967
Homsap TRBJ2-7 F ORF	0.00597	0.36418	0.31045	0.31045	0.30599	0.26756	**0.03642**	0.17103
Homsap TRBV4-1 F (F)	0.00940	0.57319	0.47922	0.47922	0.4378	0.38214	0.05008	0.23518
Homsap TRBV6-4 F	0.01023	0.62418	0.51163	0.51163	0.46602	0.40206	0.05008	0.23518
Homsap TRBV11-2 F [F] (F)	0.01067	0.65102	0.52295	0.52295	0.48031	0.40889	0.05008	0.23518
Homsap TRBJ1-5 F	0.01314	0.80141	0.63061	0.62678	0.55368	0.46996	0.05423	0.25469
Homsap TRBV12-4 F (F)	0.01334	0.81348	0.63061	0.62678	0.55911	0.46996	0.05423	0.25469
Homsap TRBV9 F (F)	0.02552	1	1	0.97616	0.79345	0.69559	0.09731	0.45700
Homsap TRBV12-3 F	0.04187	1	1	0.97616	0.92639	0.85407	0.15023	0.70553
**CD4^-^(MID4) ∼ CD4^+^(MID5) [d3]**								
Homsap TRBV5-1 F (F)	1.00E-15	**5.70E-14**	**5.70E-14**	**5.70E-14**	**5.90E-14**	**5.90E-14**	**5.70E-14**	**2.64E-13**
Homsap TRBV27 F	8.72E-13	**5.14E-11**	**5.06E-11**	**5.06E-11**	**5.14E-11**	**5.06E-11**	**2.03E-11**	**9.49E-11**
Homsap TRBV7-2 F (F)	1.03E-12	**6.10E-11**	**5.90E-11**	**5.90E-11**	**6.10E-11**	**5.90E-11**	**2.03E-11**	**9.49E-11**
Homsap TRBJ2-7 F ORF	1.32E-08	**7.76E-07**	**7.37E-07**	**7.37E-07**	**7.76E-07**	**7.37E-07**	**1.94E-07**	**9.05E-07**
Homsap TRBV7-9 F (F)	5.45E-08	**3.22E-06**	**3.00E-06**	**3.00E-06**	**3.22E-06**	**3.00E-06**	**6.43E-07**	**3.00E-06**
Homsap TRBV4-1 F (F)	7.79E-08	**4.60E-06**	**4.21E-06**	**4.21E-06**	**4.60E-06**	**4.21E-06**	**7.66E-07**	**3.57E-06**
Homsap TRBJ2-5 F	9.18E-08	**5.41E-06**	**4.86E-06**	**4.86E-06**	**5.41E-06**	**4.86E-06**	**7.73E-07**	**3.61E-06**
Homsap TRBV4-3 F (F)	8.52E-05	**0.00503**	**0.00443**	**0.00443**	**0.00502**	**0.00442**	**0.00063**	**0.00293**
Homsap TRBJ2-1 F	0.00041	**0.02424**	**0.02095**	**0.02095**	**0.02395**	**0.02074**	**0.00269**	**0.01256**
Homsap TRBV20-1 F (F)	0.00061	**0.03602**	**0.03052**	**0.03052**	**0.03539**	**0.03007**	**0.0036**	**0.0168**
Homsap TRBV15 F (F)	0.00088	0.05199	**0.04317**	**0.04317**	0.05068	**0.04227**	**0.00473**	**0.02204**
Homsap TRBV7-6 F (F)	0.00190	0.11211	0.09121	0.09121	0.10615	0.08725	**0.00934**	**0.04356**
Homsap TRBV12-4 F (F)	0.00288	0.17004	0.13545	0.13545	0.15657	0.12685	**0.01308**	0.06099
Homsap TRBV24-1 F	0.00476	0.28063	0.21880	0.21880	0.24520	0.19694	**0.02005**	0.09348
Homsap TRBJ1-5 F	0.01167	0.68846	0.52509	0.52509	0.49968	0.41032	**0.04501**	0.20991
Homsap TRBJ2-2 F	0.01221	0.72022	0.53711	0.53711	0.51551	0.41749	**0.04501**	0.20991
Homsap TRBV9 F (F)	0.01517	0.89520	0.65244	0.65244	0.59427	0.48182	0.05266	0.24556
Homsap TRBV18 F	0.01726	1	0.72497	0.72497	0.64203	0.51872	0.05658	0.26384
Homsap TRBV25-1 F	0.01860	1	0.76243	0.76243	0.66961	0.53681	0.05775	0.26928
Homsap TRBJ1-3 F	0.02756	1	1	0.98127	0.80770	0.67299	0.08129	0.37909
Homsap TRBV14 F (F)	0.03018	1	1	0.98127	0.83607	0.69739	0.0848	0.39546
Homsap TRBD2 F	0.04008	1	1	0.98127	0.91049	0.78868	0.10281	0.47944
Homsap TRBD1 F	0.04008	1	1	0.98127	0.91049	0.78868	0.10281	0.47944
Homsap TRBV12-3 F	0.04966	1	1	0.98127	0.95046	0.84016	0.12207	0.56926
**CD4^-^(MID7) ∼ CD4^+^(MID8) [d8]**								
Homsap TRBV27 F	1.36E-13	**8.04E-12**	**8.04E-12**	**8.04E-12**	**8.04E-12**	**8.04E-12**	**8.04E-12**	**3.75E-11**
Homsap TRBJ2-7 F ORF	6.45E-10	**3.80E-08**	**3.74E-08**	**3.74E-08**	**3.80E-08**	**3.74E-08**	**1.42E-08**	**6.64E-08**
Homsap TRBV5-1 F (F)	7.24E-10	**4.27E-08**	**4.13E-08**	**4.13E-08**	**4.27E-08**	**4.13E-08**	**1.42E-08**	**6.64E-08**
Homsap TRBV4-1 F (F)	6.44E-08	**3.80E-06**	**3.60E-06**	**3.60E-06**	**3.80E-06**	**3.60E-06**	**9.49E-07**	**4.43E-06**
Homsap TRBV7-2 F (F)	8.27E-07	**4.88E-05**	**4.55E-05**	**4.55E-05**	**4.88E-05**	**4.55E-05**	**9.76E-06**	**4.55E-05**
Homsap TRBJ2-5 F	1.08E-05	**0.00064**	**0.00058**	**0.00058**	**0.00064**	**0.00058**	**0.00011**	**5.00E-04**
Homsap TRBJ2-1 F	0.00024	**0.0142**	**0.01275**	**0.01275**	**0.0141**	**0.01267**	**0.00203**	**0.00946**
Homsap TRBV7-9 F (F)	5.00E-04	**0.02948**	**0.02598**	**0.02598**	**0.02906**	**0.02565**	**0.00369**	**0.01718**
Homsap TRBJ2-3 F	0.00150	0.08825	0.07628	0.07628	0.08453	0.0735	**0.00981**	**0.04573**
Homsap TRBJ1-1 F	0.00212	0.1249	0.10585	0.10585	0.11754	0.10054	**0.01249**	0.05825
Homsap TRBJ2-2 F	0.00471	0.27763	0.23057	0.23057	0.24292	0.20635	**0.02524**	0.11769
Homsap TRBV25-1 F	0.00914	0.53897	0.43848	0.43848	0.41809	0.35628	**0.04491**	0.20944
Homsap TRBV4-3 F (F)	0.01085	0.64019	0.50998	0.50998	0.47465	0.40116	**0.04925**	0.22964
Homsap TRBV5-4 F (F)	0.01423	0.83981	0.65477	0.64053	0.5708	0.48287	0.05599	0.26108
Homsap TRBV7-7 F (F)	0.01423	0.83981	0.65477	0.64053	0.5708	0.48287	0.05599	0.26108
Homsap TRBV9 F (F)	0.01776	1	0.78139	0.78139	0.65257	0.54544	0.06346	0.29591
Homsap TRBV20-1 F (F)	0.01828	1	0.78622	0.78622	0.66336	0.54774	0.06346	0.29591
Homsap TRBV12-3 F	0.02632	1	1	0.87592	0.79274	0.67382	0.08628	0.40233
Homsap TRBV18 F	0.04074	1	1	0.87592	0.91403	0.81825	0.1265	0.58987
**CD4^-^(MID10) ∼ CD4^+^(MID11) [d26]**								
Homsap TRBV27 F	2.14E-26	**1.31E-24**	**1.31E-24**	**1.31E-24**	**1.27E-24**	**1.23E-24**	**1.31E-24**	**6.14E-24**
Homsap TRBJ2-7 F ORF	4.54E-17	**2.77E-15**	**2.72E-15**	**2.72E-15**	**2.78E-15**	**2.68E-15**	**1.38E-15**	**6.50E-15**
Homsap TRBV7-2 F (F)	1.70E-14	**1.06E-12**	**1.03E-12**	**1.03E-12**	**1.06E-12**	**1.03E-12**	**3.54E-13**	**1.66E-12**
Homsap TRBV5-1 F (F)	9.10E-14	**5.54E-12**	**5.26E-12**	**5.26E-12**	**5.53E-12**	**5.26E-12**	**1.38E-12**	**6.50E-12**
Homsap TRBV4-1 F (F)	2.41E-10	**1.47E-08**	**1.38E-08**	**1.38E-08**	**1.47E-08**	**1.38E-08**	**2.94E-09**	**1.38E-08**
Homsap TRBV4-3 F (F)	1.66E-07	**1.01E-05**	**9.31E-06**	**9.31E-06**	**1.01E-05**	**9.31E-06**	**1.69E-06**	**7.94E-06**
Homsap TRBJ2-1 F	3.15E-07	**1.92E-05**	**1.73E-05**	**1.73E-05**	**1.92E-05**	**1.73E-05**	**2.74E-06**	**1.29E-05**
Homsap TRBJ2-5 F	5.49E-07	**3.35E-05**	**2.97E-05**	**2.97E-05**	**3.35E-05**	**2.97E-05**	**4.19E-06**	**1.97E-05**
Homsap TRBV20-1 F (F)	6.34E-07	**3.87E-05**	**3.36E-05**	**3.36E-05**	**3.87E-05**	**3.36E-05**	**4.30E-06**	**2.02E-05**
Homsap TRBV6-2 F (P)	1.17E-06	**7.15E-05**	**6.09E-05**	**6.09E-05**	**7.15E-05**	**6.09E-05**	**6.84E-06**	**3.21E-05**
Homsap TRBV25-1 F	1.23E-06	**7.53E-05**	**6.29E-05**	**6.29E-05**	**7.53E-05**	**6.29E-05**	**6.84E-06**	**3.21E-05**
Homsap TRBJ2-3 F	0.00005	**0.00289**	**0.00237**	**0.00237**	**0.00288**	**0.00236**	**0.00024**	**0.00113**
Homsap TRBV12-4 F (F)	0.00011	**0.00667**	**0.00536**	**0.00536**	**0.00665**	**0.00534**	**0.00051**	**0.00241**
Homsap TRBV18 F	0.00048	**0.02900**	**0.02282**	**0.02282**	**0.02859**	**0.02256**	**0.00207**	**0.00973**
Homsap TRBJ1-4 F	0.00080	**0.04853**	**0.03739**	**0.03739**	**0.04739**	**0.03672**	**0.00324**	**0.01519**
Homsap TRBV6-1 F	0.00154	0.09386	0.07078	0.07078	0.08965	0.06838	**0.00587**	**0.02755**
Homsap TRBV7-9 F (F)	0.00301	0.18336	0.13527	0.13320	0.16777	0.12670	**0.01026**	**0.04818**
Homsap TRBV7-6 F (F)	0.00303	0.18467	0.13527	0.13320	0.16885	0.12670	**0.01026**	**0.04818**
Homsap TRBV13 F (F)	0.00666	0.40628	0.28640	0.28640	0.33478	0.24975	**0.02138**	0.10042
Homsap TRBV12-3 F	0.01313	0.80117	0.55162	0.55162	0.55357	0.42609	**0.04006**	0.18812
Homsap TRBV29-1F (F)	0.01522	0.92828	0.62393	0.62393	0.60758	0.46673	**0.04420**	0.20759
Homsap TRBJ1-2 F	0.02597	1.00000	1.00000	0.98697	0.79914	0.65096	0.07201	0.33818
Homsap TRBV11-2 F [F] (F)	0.02979	1.00000	1.00000	0.98697	0.84195	0.69256	0.07901	0.37105
Homsap TRBV6-4 F	0.03543	1.00000	1.00000	0.98697	0.88923	0.74607	0.08710	0.40904
Homsap TRBV5-4 F (F)	0.03570	1.00000	1.00000	0.98697	0.89109	0.74607	0.08710	0.40904
Homsap TRBV9 F (F)	0.04172	1.00000	1.00000	0.98697	0.92569	0.78435	0.09505	0.44637
Homsap TRBJ1-3 F	0.04207	1.00000	1.00000	0.98697	0.92733	0.78435	0.09505	0.44637

Unadjusted *p*-values significant at 5% (< 0.05) are reported. Adjusted *p*-values significant at 5% (< 0.05) under the given controlling procedure are highlighted in bold.

The functionality is defined for each allele of a gene according to the rules described in http://www.imgt.org/IMGTScientificChart/SequenceDescription/IMGTfunctionality.html and can be functional, ORF or pseudogene.

- F (Functional) if the coding region has an open reading frame without stop codon, and if there is no described defect in the splicing sites, recombination signals and/or regulatory elements.

- ORF (Open Reading Frame) if the coding region has an open reading frame.

- P (Pseudogene) if the coding region has stop codon(s) and/or frameshift mutation(s).

Note that 6 pseudogenes of the TRBV group (TRBV1, TRBV3-2, TRBV12-1, TRBV12-2, TRBV21-1 and TRBV26), that cannot give productive TR chains, were excluded from the study.

In the table the functionality of all known alleles of each gene is reported and shown as follows:

- between parentheses, (F) and (P), when the accession number refers to rearranged genomic DNA or cDNA and the corresponding germline gene has not yet been isolated.

- between brackets, [F] and [P], when the accession number refers to genomic DNA, but not known as being germline or rearranged.

The importance of adjusted *p*-values can be illustrated with the example of the set comparison CD4^-^(MID1) ∼ CD4^+^(MID2) (Pre) (Table 3), where 17 differences in proportions of IMGT clonotypes (AA) are declared to be significant before controlling the error rate (unadjusted *p*-values (rawp) column), whereas only six are retained by all adjusting procedures (*p*-values < 0.05 and highlighted in bold). Differences in proportions for four additional genes are retained by BH (FDR controlling procedure). Similarly, in the set comparison CD4^-^(MID4) ∼ CD4^+^(MID5) at d3, 24 differences in proportions are declared to be significant before adjustment of *p*-values, whereas only 10 are retained by all multiple testing procedures (Table 3). Differences in proportions for one additional gene (TRBV15) is similarly retained by Holm, Hochberg, ŠidákSD, BH and BY procedures, whereas more genes are retained by BH and BY (one and four, respectively) in line with the FDR procedures being more powerful than the FWER procedures (Table 3).

### Visualization plots

**Fig 2:**
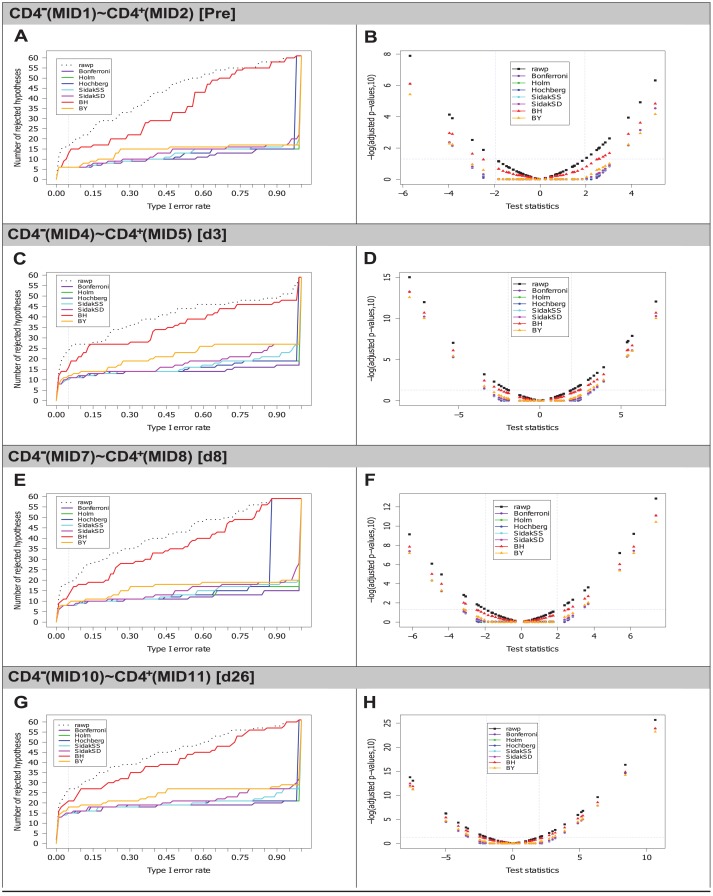
Multiple testing procedures visualization plots. Multiple testing procedures visualization plots are displayed for comparison of the differences in proportions for IMGT clonotypes (AA) with a gene of a given group (TRBV, TRBD or TRBJ), between two T cell populations (CD4^-^ and CD4^+^) at four time points (Pre, d3, d8 and d26). The following procedures: Bonferroni, Holm, Hochberg, ŠidákSS and ŠidákSD, BH and BY were applied. Left panel (A, C, E, G): Line graphs showing the number of rejected null hypotheses against the Type I error rate. Dotted lines represent unadjusted *p*-values (rawp) whereas colored lines represent ajusted *p*-values of the seven procedures. A vertical line corresponds to a Type I error rate (*α*-level) at 0.05 (significance level of 5%). Right panel (B, D, F, H): Negative decimal logarithms (-log_10_) of unadjusted *p*-values (black symbols) and adjusted *p*-values (colored symbols) against the test statistic *z*-scores. Two areas in scatter plots (top left and top right) correspond to significant differences in proportions and they are delimited at a significance level of 5% (0.05) by -log_10_(*p*-values) > 1.3 (horizontal line) and by *z*-scores (< -1.96 for negative differences or > 1.96 for positive differences) (vertical line).

In order to visualize the statistical analysis results obtained using the multiple testing procedures, two types of plots (line graphs and scatter plots, Fig 2) were generated for each comparison using the Bioconductor (http://www.bioconductor.org) R package multtest [38].

The line graphs (Fig 2, left panel) represent the number of rejected hypotheses (number of significant values in Table 3) against the Type I error rate (*α*-level). Dotted lines represent unadjusted *p*-values (rawp) whereas colored lines represent ajusted *p*-values of the seven procedures. These graphs allow to visualize the number of rejected null hypotheses for a chosen *α*-level under a given procedure. Thus, by fixing the smallest adjusted *p*-values obtained by the procedures at a Type I error rate (*α*-level = 0.05) (vertical line in Fig 2), the numbers of rejected null hypotheses (numbers of adjusted *p*-values in bold in Table 3), for example with the BH procedure (red line), are 10 (Pre), 16 (d3), 13 (d8) and 21 (d26), whereas with the Bonferroni procedure (purple line), they are 6 (Pre), 10 (d3), 8 (d8) and 15 (d26). The line graphs also allow to identify, for a selected number of rejected null hypotheses, the *α*-level which is required to get that number for a given procedure.

The scatter plots (Fig 2, right panel) display negative decimal logarithms (-log_10_) of unadjusted *p*-values (black symbols) and adjusted *p*-values obtained by each multiple testing procedure (colored symbols) against the test statistics *z*-scores on abscissa (values in S2 Table).

As the *p*-values range from 0 to 1, their negative decimal logarithms are used in the plots to provide a larger visual representation. Thus, a *p*-value of 0.05 corresponds to -log_10_(0.05) ≃ 1.3 (horizontal line in Fig 2). Therefore, significant differences in proportions of IMGT clonotypes (AA) with a gene *k* at a Type I error rate *α*-level = 0.05 for the different procedures correspond to those represented with a -log_10_(*p*-value) > 1.3 and with a *z*-score less than -1.96 (< -1.96) for negative differences or with a *z*-score greater than 1.96 (> 1.96) for positive differences. Differences are negative if the proportion of IMGT clonotypes (AA) is greater in the second set (CD4^+^) than in the first set (CD4^-^) ($p_{\left\{{\text{CD4}}^{-}\right\}}^{k}<p_{\left\{{\text{CD4}}^{+}\right\}}^{k}$) and, alternatively, differences are positive if the proportion of the IMGT clonotypes (AA) is greater in the first set (CD4^-^) than in the second set (CD4^+^) ($p_{\left\{{\text{CD4}}^{-}\right\}}^{k}>p_{\left\{{\text{CD4}}^{+}\right\}}^{k}$).

The genes with the highest significant differences in proportion of IMGT clonotypes (AA) between the two T cell populations CD4^-^ and CD4^+^ are in the top left ($p_{\left\{{\text{CD4}}^{-}\right\}}^{k}<p_{\left\{{\text{CD4}}^{+}\right\}}^{k}$) and top right ($p_{\left\{{\text{CD4}}^{-}\right\}}^{k}>p_{\left\{{\text{CD4}}^{+}\right\}}^{k}$) of each scatter plot (Fig 2, right panel) and can be identified in S2 Table. For examples, the three genes identified with highly significant negative difference in proportions for CD4^-^(MID1) ∼ CD4^+^(MID2) (Pre) are: TRBV5-1 (*z*-score = -5.686, -log_10_(rawp) = 7.886, -log_10_(adjusted *p*-values) = 6.101 (FWER procedure and BH) and 5.429 (BY)), TRBV7-2 (*z*-score = -3.965, -log_10_(rawp) = 4.135, log_10_(adjusted *p*-values) from 2.350 to 2.373 (FWER), 2.952 (BH) and 2.280 (BY)) and TRBV20-1 (*z*-score = -3.837, -log_10_(rawp) = 3.904, -log_10_(adjusted *p*-values) from 2.119 to 2.157 (FWER), 2.897 (BH) and 2.225 (BY)) (S2 Table and top left of Fig 2B). The three genes identified with the highly significant positive differences for CD4^-^(MID1) ∼ CD4^+^(MID2) (Pre) are: TRBV27 (*z*-score = 5.034, -log_10_(rawp) = 6.318, -log_10_(adjusted *p*-values) from 4.532 to 4.540 (FWER procedures), 4.833 (BH) and 4.162 (BY)), TRBJ2-1 (*z*-score = 4.378, -log_10_(rawp) = 4.922, log_10_(adjusted *p*-values) from 3.136 to 3.151 (FWER), 3.613 (BH) and 2.942 (BY)) and TRBV7-9 (*z*-score = 3.858, -log_10_(rawp) = 3.942, -log_10_(adjusted *p*-values) from 2.157 to 2.187 (FWER), 2.897 (BH) and 2.225 (BY)) (S2 Table and top right of Fig 2B).

### Difference in proportions graph

**Fig 3:**
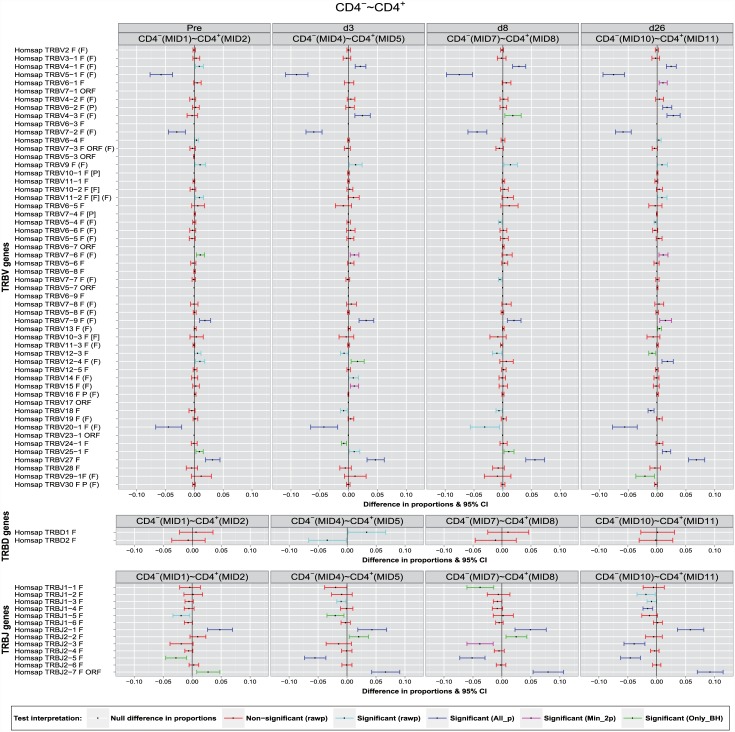
Differences in proportions graph. The difference in proportions graph with significance and confidence interval (CI) bars are shown for *Homo sapiens* TRB IMGT clonotypes (AA) with a gene of a given group (TRBV, TRBD or TRBJ) between two T cell populations (CD4^-^ and CD4^+^) at four time points (Pre, d3, d8 and d26). Negative differences in proportions (*p*_{CD4^−^}_-*p*_{CD4^+^}_ < 0) are shown on the left of the vertical line (abscissa equal to 0) and positive differences (*p*_{CD4^−^}_-*p*_{CD4^+^}_ > 0) are shown on the right of the vertical line. CI bars colors indicated in the legend correspond to the test interpretation before adjustment of *p*-values (rawp) (significant differences validated by the seven procedures (All_p): dark blue, by two or more multiple testing procedures (Min_2p): pink, and only by BH (Only_BH): green).

The difference in proportions graph (Fig 3) shows the differences in proportions with significance and confidence intervals (CI) of IMGT clonotypes (AA) with a gene *k* of a given group (TRBV, TRBD or TRBJ) between the two T cell populations (CD4^-^ and CD4^+^) at the four time points (Pre, d3, d8 and d26). The point in the middle of each 95% CI bar corresponds to the value of the difference in proportions of IMGT clonotypes (AA) with a gene *k* found in two different sets. Differences in proportions and 95% CI lower and upper bounds are reported in S1 Table for all cases with unadjusted p-values < 0.05 of Table 3. Negative differences in proportions of the IMGT clonotypes (AA) (proportion greater in the second set (CD4^+^) than in the first set (CD4^-^)) are displayed on the left of the vertical reference line (abscissa equal to 0). Alternatively, positive differences in proportions of the IMGT clonotype (AA) (proportion greater in the first set (CD4^-^) than in the second set (CD4^+^)) are displayed on the right of the vertical line. CI bars are colored based on the interpretation test before adjustment (legend in Fig 3): red for non-significant difference (i.e., -1.96 < z-score < 1.96) or light blue for significant difference (i.e., z-score < -1.96 or z-score > 1.96). If a CI bar does not contain 0 (i.e., does not cross the vertical reference line), this is a clear indication that there is a statistically significant difference. More the point in the middle of a CI bar is far from the vertical reference line, more the significance of the difference in proportions is important. Lower levels of variance yield shorter CI bar widths, and therefore more accurate estimates.

Considering the results of multiple testing procedures, the CI bars declared as significant by the seven adjustment procedures (Table 3) are colored in dark blue. If the significance is validated by two or more multiple testing procedures, CI bars are colored in pink. If the significance is validated only by the BH procedure, CI bars are colored in green.

As examples, the negative differences in proportions of the IMGT clonotypes (AA) of the TRBV5-1 (-0.057247), TRBV7-2 (-0.030195) and TRBV20-1 (-0.044506) genes (S1 Table) between sets CD4^-^ and CD4^+^ at Pre are displayed on the left of the vertical reference line in panel Pre(MID1) ∼ Pre(MID2) (Fig 3) (${\text{p}}_{\left\{{\text{CD4}}^{-}\right\}}^{k}<p_{\left\{{\text{CD4}}^{+}\right\}}^{k}$). The positive difference in proportions of IMGT clonotypes (AA) of the TRBV7-9 (+0.018678), TRBV27 (+0.031817) and TRBJ2-1 (+0.048132) are displayed on the right of the vertical line (${\text{p}}_{\left\{{\text{CD4}}^{-}\right\}}^{k}>p_{\left\{{\text{CD4}}^{+}\right\}}^{k}$). The CI bars of these 6 genes colored in dark blue indicate that the significance was validated by all procedures (i.e., adjusted *p*-values < 0.05, Table 3). In the same panel, the CI bars of TRBV7-6, TRBV25-1, TRBJ2-5 and TRBJ2-7 colored in green indicate that the significance is validated by only the BH procedure (Table 3). The CI bars colored in pink, present in the other panels, indicate that the significance of the difference in proportions is validated by two or more procedures, for example, TRBV7-6 gene at d3 validated by the BH (*P* = 0.009) and the BY procedures (*P* = 0.043) (Table 3, d3) and (Fig 3: ‘d3’ panel).

### Synthesis graph

**Fig 4:**
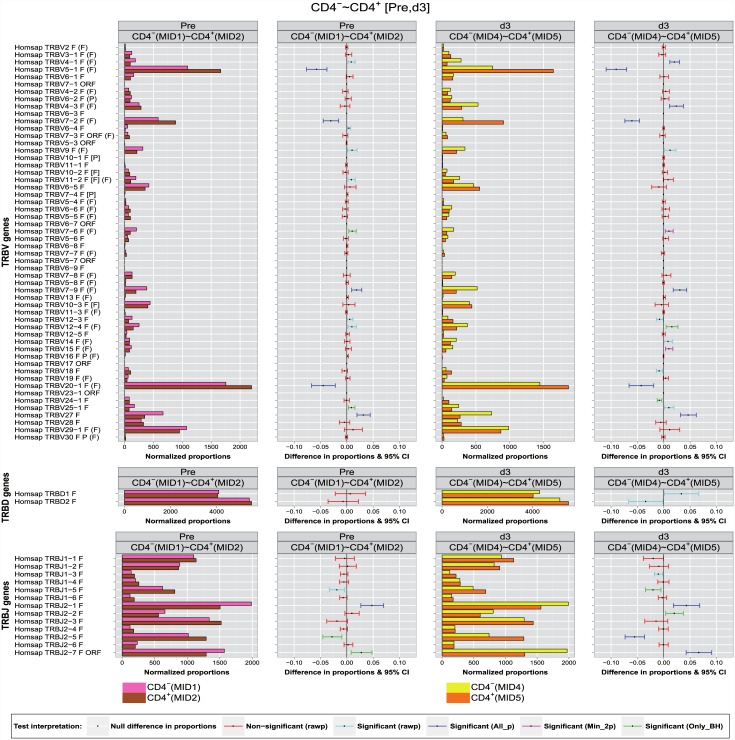
Synthesis graph (part 1: Pre, d3). The synthesis graph is displayed for Homo sapiens TRB IMGT clonotypes (AA) with a gene of a given group (TRBV, TRBD or TRBJ) between two T cell populations (CD4^-^ and CD4^+^) at two time points (Pre and d3), with for each time point, two panels. Left panel: normalized bar graph (IMGT clonotypes (AA) proportions normalized for 10,000 IMGT clonotypes (AA) per group), with juxtaposed colored bars corresponding to CD4^-^ (top) and CD4^+^ (bottom). Right panel: difference in proportions graph with significance and confidence interval (CI) bars. CI bar colors correspond to the test interpretation before adjustment of *p*-values (rawp) (non-significant: red, significant: light blue) and after adjustment by the multiple testing procedures (significant differences validated by the seven procedures (All_p): dark blue, by two or more multiple testing procedures (Min_2p): pink, and only by BH (Only_BH): green).

**Fig 5:**
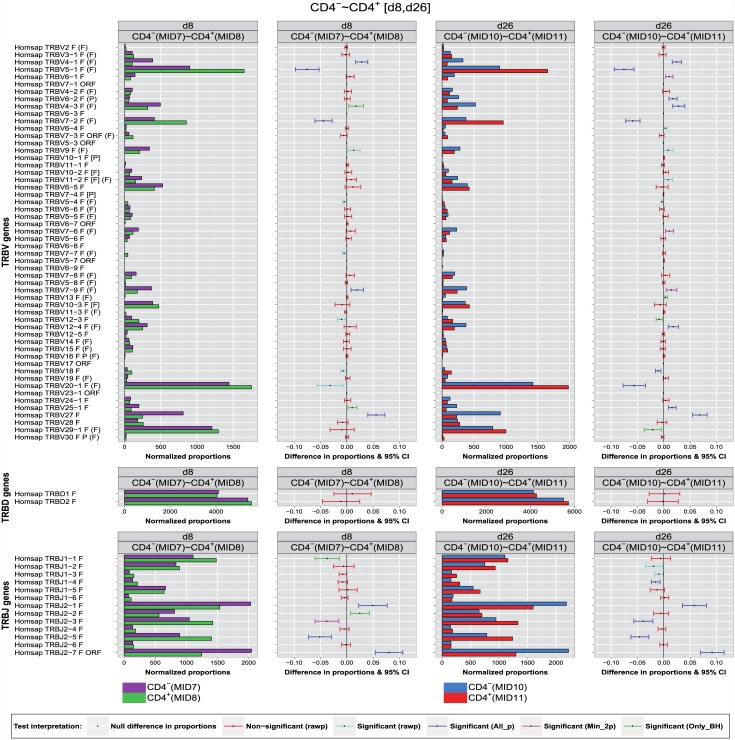
Synthesis graph (part 2: d8, d26). The synthesis graph is displayed for Homo sapiens TRB IMGT clonotypes (AA) with a gene of a given group (TRBV, TRBD or TRBJ) between two T cell populations (CD4^-^ and CD4^+^) at two time points (d8 and d26), with for each time point, two panels. Left panel: normalized bar graph (IMGT clonotypes (AA) proportions normalized for 10,000 IMGT clonotypes (AA) per group), with juxtaposed colored bars corresponding to CD4^-^ (top) and CD4^+^ (bottom). Right panel: difference in proportions graph with significance and confidence interval (CI) bars. CI bar colors correspond to the test interpretation before adjustment of *p*-values (rawp) (non-significant: red, significant: light blue) and after adjustment by the multiple testing procedures (significant differences validated by the seven procedures (All_p): dark blue, by two or more multiple testing procedures (Min_2p): pink, and only by BH (Only_BH): green).

To facilitate the comparison with the experimental results, a synthesis graph (Fig 4) is proposed that combines the display, in parallel panels, of the normalized bar graphs of the proportions and the differences in proportions with significance and confidence intervals (CI bars), described in the previous sections. A few examples are given to illustrate the interpretation and comparisons (Figs 4 and 5).

The important differences in proportions of IMGT clonotypes (AA) of the TRBV5-1, TRBV7-2, TRBV20-1 and TRBJ2-5 genes with higher clonotype diversity in CD4^+^ compared to CD4^-^ at the four time points (Pre, d3, d8, d26) was validated by all multiple testing procedures at an *α*-level = 0.05 (Table 3) (Fig 4) (dark blue CI bars) with the exception of TRBV20-1 at d8 (only validated before adjustment: unadjusted *p*-value = 0.01, light blue CI bar) and of TRBJ2-5 (Pre) where the significance was validated only by the BH method (*P* = 0.023, Table 3) and Fig 4 (green CI bar in ‘Pre’ panel).

The higher clonotype diversity of the TRBJ2-7 gene in CD4^-^ compared to CD4^+^ observed before vaccination (Pre), at d3, d8 and d26 post-vaccination was validated by all multiple testing procedures (*P* < 10^−6^, Table 3) (Fig 4, dark blue CI bar), whereas before vaccination (Pre) it is validated only by the BH procedure (*P* = 0.036, Table 3).

The slight differences in proportions of the IMGT clonotypes (AA) of the TRBV4-1 and TRBV4-3 genes were declared as significant at d3, d8 and d26 post-vaccination by all adjustment methods, except for TRBV4-3 at day 8, when the significance was validated only by the BH procedure. Slight significant differences in clonotype diversity were also observed at the four time points for TRBV7-9 but only declared significant at Pre, d3, d8 by all adjustment methods and at d26 by the BH and BY procedures. For TRBV12-4 gene, the significance of the clonotype diversity was validated by all adjusting procedure at d26, but only by the BH procedure at d3. For TRBJ2-2 gene at d3 and d8 post-vaccination, differences in proportions were declared as significant only by the BH procedure.

A potentially important finding by comparing CD4^-^ and CD4^+^ populations is the fact that the vaccination did not change the sign of significant differences in proportions found before vaccination. In others words, if the proportion of IMGT clonotypes (AA) having a specific gene *k* in CD4^-^ exceeds that in CD4^+^ at baseline (i.e., *p*_{CD4^−^}_-*p*_{CD4^+^}_ > 0), this positive sign remains unchanged until d26. The repertoire characteristics in terms of IMGT clonotype diversity (AA) per gene is therefore kept at any given time point and the differences in proportionsobserved are maintained between the two T cell populations CD4^-^ and CD4^+^.

## Discussion

In this study, we define a standardized IMGT/HighV-QUEST procedure for the evaluation of the significance of the IMGT clonotypes (AA) diversity differences in proportions between NGS immunoprofiles. We statistically analyzed the diversity of IMGT clonotypes (AA) of each gene of a given group (TRBV, TRBD or TRBJ) between two T cells populations (CD4^-^ and CD4^+^) at four time points pre-vaccination (Pre) and d3, d8 and d26 post-vaccination of a single individual vaccinated against H1N1 influenza virus. We first applied a test statistic *z*-score using a weighted pooled proportion to compare the IMGT clonotypes (AA) diversity of each gene of a given group between sets.

To control introduced errors when testing many null hypotheses, we applied FWER (Bonferroni, Holm, Hochberg, ŠiddákSS and Šidák SD) and FDR (BH and BY) controlling procedures. We observed that FWER procedures, and especially Bonferroni, Holm and Hochberg, gave very similar adjusted *p*-values (i.e., same number of rejected null hypotheses). This is most likely due to the independence structure of the unadjusted *p*-values [30, 34]. We also noted that the FDR (BY and especially BH) procedures gave more rejected null hypotheses confirming that they are more powerful and less conservative than the FWER procedures.

These observations are important for the choice of the controlling procedures for multiple significance testing as shown in this study for the evaluation of the statistical significance of IMGT clonotypes (AA) diversity per gene. FWER controlling procedures (Bonferroni, Holm, Hochberg, ŠidákSS and ŠidákSD) have been criticized for genomics studies as being very stringent methods by avoiding Type I errors and therefore leading to loss of information in some treated cases with many Type II errors (*β*-level) (i.e., when the statistical test of the hypotheses leads to accept a null hypothesis $H_{0}^{k}$ when it is false) [30]. Bonferroni, the most popular method, is also the most conservative one according to several simulation studies [39, 40], however, it is valid even if the test statistics are dependent. Šidák (ŠidákSS), a single-step procedure based on the Bonferroni method, is used in the case of independently distributed unadjusted *p*-values and it does not ensure a good control for random distributions of the test statistics [34]. Šidák (ŠidákSD), a step-down procedure is developed to improve the conservative results of single-step methods. Holm, a step-down procedure, is also considered as less conservative than the Bonferroni method and it makes no hypotheses on the dependence of the unadjusted *p*-values. Hochberg, a step-up modification of the Holm procedure, is more powerful than the Bonferroni correction and makes assumptions on the dependence structure of the unadjusted *p*-values. Our study confirms a similarity in the results found by using the Bonferroni, Holm, Hochberg, ŠiddákSS and Šidák SD procedures in terms of the number of rejected null hypotheses.

The improvements of other procedures for corrections of multiple comparison tests following the Bonferroni procedure focus, in general, on the dependence or ignorance of test statistics under null hypotheses. Despite the assumption of the independence between IMGT clonotypes (AA) with a gene *k* of a given group in this study (i.e., independence of unadjusted *p*-values), strong consideration will be given to results validated by all procedures to keep a Type I error control as reliable as possible.

Our study confirms that the control of the FDR is more powerful than the control of the FWER, with the BH method being even less conservative than the BY procedure as previously shown [28, 30, 34, 39]. For this reason, a special consideration should be given to results obtained by the BH procedure.

IMGT/HighV-QUEST represents a major breakthrough for the NGS analysis and the comparison of the antigen receptor V domain repertoires and immunoprofiles of the adaptive immune response [9, 15]. Indeed, IMGT/HighV-QUEST provides standardized and high-quality outputs based on the IMGT-ONTOLOGY concepts of identification, description, classification and numerotation [2]. IMGT/HighV-QUEST comprises more than 500 features per sequence allowing a detailed characterization of the antigen receptor (IG and TR) V domain repertoires. Comparison between batches up to 1 million sequences can be performed online. The analysis of these repertoires are invaluable for the comparison of immune profiles in protective (vaccination, infections, cancers) or pathogenic (autoimmunity, lymphoproliferative disorders) immune responses [41].

One main feature of immunoprofiles is the comparison of the IMGT clonotypes (AA) diversity and expression. The IMGT/HighV-QUEST statistical procedure implemented for evaluating the significance of the IMGT clonotypes (AA) adds a further major step in the repertoire analysis. Based on the IMGT-ONTOLOGY concepts, the analysis is generic. It is applicable to the IMGT clonotypes (AA) diversity of the four TR loci (TRA, TRB, TRG, TRD) [5] and of the three IG loci (IGH, IGK, IGL) [4]. IMGT/HighV-QUEST outputs provide the number of sequences per IMGT clonotype (AA) (IMGT clonotype expression) based on the same standardized criteria and IMGT-ONTOLOGY concepts [16]. The procedure is therefore suitable for evaluating significance of the IMGT clonotype (AA) diversity as well as the significance of IMGT clonotype (AA) expression changes of a gene for a given group. More generally, this procedure can also be used for statistical comparison of IMGT clonotypes (AA) of different B or T cell populations in order to detect significant changes in immunoprofiles observed in normal or pathological situations, in one individual or between individuals.

## Supporting Information

S1 Table Number (nb) of *Homo sapiens* TRB IMGT clonotypes (AA) and normalized nb for 10,000. The number (nb) of *Homo sapiens* TRB IMGT clonotypes (AA) and normalized nb for 10,000 per gene and per group (TRBV, TRBD or TRBJ), differences in proportions and corresponding 95% confidence intervals (CI) are given between two T cell populations (CD4^-^ and CD4^+^) at four time points (Pre, d3, d8 and d26).
(PDF)

S2 Table Test statistics (*z*-scores) and negative decimal logarithms (-log_10_). The test statistics (*z*-scores) and negative decimal logarithms (-log_10_) for unadjusted (rawp) and adjusted *p*-values for *Homo sapiens* TRB IMGT clonotypes (AA) with a gene of a given group (TRBV, TRBD or TRBJ) are given between two T cell populations (CD4^-^ and CD4^+^) at four time points (Pre, d3, d8 and d26).
(PDF)
