# Correction: IMGT/HighV-QUEST Statistical Significance of IMGT Clonotype (AA) Diversity per Gene for Standardized Comparisons of Next Generation Sequencing Immunoprofiles of Immunoglobulins and T Cell Receptors

**DOI:** 10.1371/journal.pone.0146702

**Published:** 2016-01-05

**Authors:** Safa Aouinti, Dhafer Malouche, Véronique Giudicelli, Sofia Kossida, Marie-Paule Lefranc

The following information is missing from the Funding section: Funding for open access charge: IMGT (Montpellier University and CNRS).

Additionally, the following text is missing from the Acknowledgements section: IMGT^®^ is currently supported by the Centre National de la Recherche Scientifique (CNRS); the Ministère de l'Enseignement Supérieur et de la Recherche (MESR); the Montpellier University, France; the Agence Nationale de la Recherche (ANR) Labex MabImprove [ANR-10-LABX-53-01]; BioCampus Montpellier; Région Languedoc-Roussillon (Grand Plateau Technique pour la Recherche (GPTR)). This work was granted access to the HPC@LR and to the High Performance Computing (HPC) resources of the Centre Informatique National de l'Enseignement Supérieur (CINES) and to Très Grand Centre de Calcul (TGCC) of the Commissariat à l'Energie Atomique et aux Energies Alternatives (CEA) under the allocation [036029] (2010–2015) made by GENCI (Grand Equipement National de Calcul Intensif). Funding for open access charge: IMGT (Montpellier University and CNRS)
