# Supplementary material for: IMGT/HighV-QUEST Statistical Significance of IMGT Clonotype (AA) Diversity per Gene for Standardized Comparisons of Next Generation Sequencing Immunoprofiles of Immunoglobulins and T Cell Receptors
Source: PLoS One. 2015 Nov 5;10(11):e0142353. doi: 10.1371/journal.pone.0142353 (PMC4634997; doi:10.1371/journal.pone.0142353)
Supplement: S1 Table — The number (nb) of Homo sapiens TRB IMGT clonotypes (AA) and normalized nb for 10,000 per gene and per group (TRBV, TRBD or TRBJ), differences in proportions and corresponding 95% confidence intervals (CI) are given between two T cell populations (CD4- and CD4+) at four time points (Pre, d3, d8 and d26). (PDF) [file pone.0142353.s001.pdf]

S1 Table. Number (nb) of *Homo sapiens* TRB IMGT clonotypes (AA) and normalized nb for 10,000. The number (nb) of *Homo sapiens* TRB IMGT clonotypes (AA) and normalized nb for 10,000 per gene and per group (TRBV, TRBD or TRBJ), differences in proportions and corresponding 95% confidence intervals (CI) are given between two T cell populations (CD4- and CD4+) at four time points (Pre, d3, d8 and d26).

Set comparisons	Nb of IMGT clonotypes (AA)		Normalized nb of IMGT clonotypes (AA) for 10,000 per group		Difference in proportions	Difference in proportions 95% CI	
	CD4− (set *i*)	CD4+ (set *j*)	CD4− (set *i*)	CD4+ (set *j*)		lower bound	upper bound
**CD4−(MID1)∼CD4+(MID2) [Pre]**							
Homsap TRBV5-1 F (F)	244	420	1092.21	1664.68	-0.057247	-0.076704	-0.037790
Homsap TRBV27 F	149	88	666.97	348.79	0.031817	0.019235	0.044399
Homsap TRBJ2-1 F	444	380	1987.47	1506.14	0.048132	0.026484	0.069780
Homsap TRBV7-2 F (F)	130	223	581.92	888.87	-0.030195	-0.044923	-0.015466
Homsap TRBV7-9 F (F)	86	50	384.96	198.18	0.018678	0.009022	0.028333
Homsap TRBV20-1 F (F)	392	555	1754.70	2199.76	-0.044506	-0.067090	-0.021921
Homsap TRBV7-6 F (F)	46	25	205.91	99.09	0.010682	0.003638	0.017725
Homsap TRBJ2-5 F	227	326	1061.11	1292.11	-0.027599	-0.045718	-0.009480
Homsap TRBV25-1 F	38	20	170.10	79.27	0.009082	0.002701	0.015464
Homsap TRBJ2-7 F, ORF	351	326	1571.17	1292.11	0.027906	0.007929	0.047882
Homsap TRBV4-1 F (F)	42	25	188.00	99.09	0.008891	0.002060	0.015722
Homsap TRBV6-4 F	12	3	53.72	11.89	0.004182	0.000866	0.007498
Homsap TRBV11-2 F [F] (F)	44	27	192.96	107.02	0.008994	0.001971	0.016017
Homsap TRBJ1-5 F	139	204	622.20	808.56	-0.018635	-0.033247	-0.004024
Homsap TRBV12-4 F (F)	56	38	250.67	150.61	0.010005	0.001967	0.018043
Homsap TRBV9 F (F)	71	54	317.82	214.03	0.010378	0.001169	0.019587
Homsap TRBV12-3 F	29	18	129.81	71.34	0.005846	0.000118	0.011575
**CD4−(MID4)∼CD4+(MID5) [d3]**							
Homsap TRBV5-1 F (F)	104	471	748.74	1647.43	-0.089868	-0.109271	-0.070466
Homsap TRBV27 F	102	76	734.34	265.83	0.046851	0.031919	0.061783
Homsap TRBV7-2 F (F)	43	260	309.58	909.41	-0.059983	-0.073913	-0.046053
Homsap TRBJ2-7 F, ORF	274	373	1972.64	1304.65	0.066799	0.042501	0.091097
Homsap TRBV7-9 F (F)	72	60	518.36	209.86	0.030849	0.018061	0.043638
Homsap TRBV4-1 F (F)	39	21	280.78	73.45	0.020733	0.011498	0.029967
Homsap TRBJ2-5 F	103	369	741.54	1290.66	-0.054912	-0.073376	-0.036448
Homsap TRBV4-3 F (F)	74	83	532.76	290.31	0.024245	0.010926	0.037563
Homsap TRBJ2-1 F	277	446	1994.24	1559.99	0.043425	0.018556	0.068295
Homsap TRBV20-1 F (F)	201	535	1447.08	1871.28	-0.042420	-0.065802	-0.019038
Homsap TRBV15 F (F)	22	16	158.39	55.96	0.010242	0.003130	0.017355
Homsap TRBV7-6 F (F)	24	20	172.79	69.95	0.010283	0.002780	0.017786
Homsap TRBV12-4 F (F)	52	62	374.37	216.86	0.015751	0.004430	0.027072
Homsap TRBV24-1 F	3	29	21.60	101.43	-0.007984	-0.012394	-0.003573
Homsap TRBJ1-5 F	68	197	489.56	689.05	-0.019949	-0.034611	-0.005287
Homsap TRBJ2-2 F	112	172	806.34	601.61	0.020473	0.003710	0.037236
Homsap TRBV9 F (F)	47	61	338.37	213.36	0.012501	0.001617	0.023386
Homsap TRBV18 F	8	40	57.60	139.91	-0.008231	-0.014094	-0.002368
Homsap TRBV25-1 F	34	41	244.78	143.41	0.010137	0.000916	0.019359
Homsap TRBJ1-3 F	17	63	122.39	220.36	-0.009797	-0.017696	-0.001898
Homsap TRBV14 F (F)	29	35	208.78	122.42	0.008636	0.000105	0.017168
Homsap TRBD2 F	724	1599	5212.38	5592.86	-0.034002	-0.066550	-0.001455
Homsap TRBD1 F	599	1152	4312.46	4029.38	0.034002	0.001455	0.066550
Homsap TRBV12-3 F	12	46	86.39	160.90	-0.007450	-0.014155	-0.000745
**CD4−(MID7)∼CD4+(MID8) [d8]**							
Homsap TRBV27 F	106	49	809.78	249.24	0.056054	0.039748	0.072360
Homsap TRBJ2-7 F, ORF	268	245	2047.36	1246.19	0.080118	0.053831	0.106405
Homsap TRBV5-1 F (F)	118	325	901.45	1653.10	-0.075165	-0.097756	-0.052575
Homsap TRBV4-1 F (F)	51	21	389.61	106.82	0.028279	0.016854	0.039705
Homsap TRBV7-2 F (F)	54	168	412.53	854.53	-0.044200	-0.060594	-0.027805
Homsap TRBJ2-5 F	117	276	893.81	1403.87	-0.051005	-0.072792	-0.029218
Homsap TRBJ2-1 F	266	302	2032.09	1536.11	0.049597	0.022593	0.076601
Homsap TRBV7-9 F (F)	49	35	374.33	178.03	0.019631	0.007802	0.031459

Homsap TRBJ2-3 F	137	280	1046.60	1424.21	-0.037761	-0.060425	-0.015097
Homsap TRBJ1-1 F	145	291	1107.72	1480.16	-0.037245	-0.060385	-0.014104
Homsap TRBJ2-2 F	106	110	809.78	559.51	0.025027	0.007093	0.042960
Homsap TRBV25-1 F	26	18	198.62	91.56	0.010707	0.002055	0.019359
Homsap TRBV4-3 F (F)	65	63	496.56	320.45	0.017611	0.003501	0.031722
Homsap TRBV5-4 F (F)	0	9	0.00	45.78	-0.004578	-0.007562	-0.001594
Homsap TRBV7-7 F (F)	0	9	0.00	45.78	-0.004578	-0.007562	-0.001594
Homsap TRBV9 F (F)	45	41	343.77	208.55	0.013523	0.001804	0.025241
Homsap TRBV20-1 F (F)	189	345	1443.85	1754.83	-0.031098	-0.056501	-0.005696
Homsap TRBV12-3 F	13	39	99.31	198.37	-0.009906	-0.018082	-0.001730
Homsap TRBV18 F	5	20	38.20	101.73	-0.006353	-0.011907	-0.000800
**$CD4^-$(MID10)∼$CD4^+$(MID11) [d26]**							
Homsap TRBV27 F	176	66	914.76	229.57	0.068520	0.054523	0.082516
Homsap TRBJ2-7 F, ORF	427	373	2219.33	1297.39	0.092194	0.069931	0.114458
Homsap TRBV7-2 F (F)	72	276	374.22	960.00	-0.058578	-0.072285	-0.044871
Homsap TRBV5-1 F (F)	174	476	904.37	1655.65	-0.075129	-0.093806	-0.056451
Homsap TRBV4-1 F (F)	63	23	327.44	80.00	0.024744	0.016151	0.033337
Homsap TRBV4-3 F (F)	101	69	524.95	240.00	0.028495	0.017066	0.039923
Homsap TRBJ2-1 F	420	460	2182.95	1600.00	0.058395	0.035485	0.081105
Homsap TRBJ2-5 F	151	356	784.82	1238.26	-0.045344	-0.062355	-0.028333
Homsap TRBV20-1 F (F)	274	570	1424.12	1982.61	-0.055849	-0.077209	-0.034489
Homsap TRBV6-2 F (P)	50	24	259.88	83.48	0.017640	0.009791	0.025488
Homsap TRBV25-1 F	44	19	228.69	66.09	0.016260	0.008953	0.023567
Homsap TRBJ2-3 F	182	383	945.95	1332.17	-0.038623	-0.056659	-0.020587
Homsap TRBV12-4 F (F)	72	55	374.22	191.30	0.018292	0.008443	0.028140
Homsap TRBV18 F	8	42	41.58	146.09	-0.010451	-0.015695	-0.005206
Homsap TRBJ1-4 F	30	89	155.93	309.57	-0.015364	-0.023774	-0.006954
Homsap TRBV6-1 F	37	25	192.31	86.96	0.010535	0.003522	0.017548
Homsap TRBV7-9 F (F)	74	68	384.62	236.52	0.014809	0.004577	0.025042
Homsap TRBV7-6 F (F)	44	34	228.69	118.26	0.011043	0.003282	0.018804
Homsap TRBV13 F (F)	10	3	51.98	10.43	0.004154	0.000731	0.007577
Homsap TRBV12-3 F	16	48	83.16	166.96	-0.008380	-0.014577	-0.002183
Homsap TRBV29-1F (F)	153	288	795.22	1001.74	-0.020652	-0.036980	-0.004324
Homsap TRBJ1-2 F	144	268	748.44	932.17	-0.018373	-0.034223	-0.002524
Homsap TRBV11-2 F [F] (F)	47	45	244.28	156.52	0.008776	0.000520	0.017033
Homsap TRBV6-4 F	10	5	51.98	17.39	0.003458	-0.000097	0.007014
Homsap TRBV5-4 F (F)	1	10	5.20	34.78	-0.002959	-0.005339	-0.000578
Homsap TRBV9 F (F)	54	55	280.67	191.30	0.008936	0.000018	0.017855
Homsap TRBJ1-3 F	32	73	166.32	253.91	-0.008759	-0.016866	-0.000652

Information is shown for differences in proportions having the smallest unadjusted p-values (<0.05, $-\log_{10} > 1.3$) as analyzed in this study.
See Table 1 for total number of IMGT clonotypes (AA) for the compared sets.
