# Supplementary material for: IMGT/HighV-QUEST Statistical Significance of IMGT Clonotype (AA) Diversity per Gene for Standardized Comparisons of Next Generation Sequencing Immunoprofiles of Immunoglobulins and T Cell Receptors
Source: PLoS One. 2015 Nov 5;10(11):e0142353. doi: 10.1371/journal.pone.0142353 (PMC4634997; doi:10.1371/journal.pone.0142353)
Supplement: S2 Table — The test statistics (z-scores) and negative decimal logarithms (-log10) for unadjusted (rawp) and adjusted p-values for Homo sapiens TRB IMGT clonotypes (AA) with a gene of a given group (TRBV, TRBD or TRBJ) are given between two T cell populations (CD4- and CD4+) at four time points (Pre, d3, d8 and d26). (PDF) [file pone.0142353.s002.pdf]

S2 Table. Test statistics (z-scores) and negative decimal logarithms (-$\log_{10}$). The test statistics (z-scores) and negative decimal logarithms (-$\log_{10}$) for unadjusted (rawp) and adjusted p-values for *Homo sapiens* TRB IMGT clonotypes (AA) with a gene of a given group (TRBV, TRBD or TRBJ) are given between two T cell populations (CD4- and CD4+) at four time points (Pre, d3, d8 and d26).

**Set comparisons**	**Test statistics (z-scores)**	**-$\log_{10}$ (unadjusted p-values (rawp))**	**-$\log_{10}$(adjusted p-values)**						
			**Bonferroni**	**Holm**	**Hochberg**	**ŠidákSS**	**ŠidákSD**	**BH**	**BY**
**CD4$^-$(MID1)∼CD4$^+$(MID2) [Pre]**									
Homsap TRBV5-1 F (F)	-5.686	7.886	**6.101**	**6.101**	**6.101**	**6.101**	**6.101**	**6.101**	**5.429**
Homsap TRBV27 F	5.034	6.318	**4.532**	**4.540**	**4.540**	**4.532**	**4.540**	**4.833**	**4.162**
Homsap TRBJ2-1 F	4.378	4.922	**3.136**	**3.151**	**3.151**	**3.137**	**3.151**	**3.613**	**2.942**
Homsap TRBV7-2 F (F)	-3.965	4.135	**2.350**	**2.372**	**2.372**	**2.351**	**2.373**	**2.952**	**2.280**
Homsap TRBV7-9 F (F)	3.858	3.942	**2.157**	**2.186**	**2.186**	**2.158**	**2.187**	**2.897**	**2.225**
Homsap TRBV20-1 F (F)	-3.837	3.904	**2.119**	**2.156**	**2.156**	**2.120**	**2.157**	**2.897**	**2.225**
Homsap TRBV7-6 F (F)	3.032	2.615	0.830	0.875	0.875	0.861	0.903	**1.675**	1.003
Homsap TRBJ2-5 F	-2.964	2.518	0.732	0.785	0.785	0.771	0.820	**1.635**	0.964
Homsap TRBV25-1 F	2.849	2.358	0.572	0.633	0.633	0.628	0.682	**1.527**	0.855
Homsap TRBJ2-7 F ORF	2.749	2.224	0.439	0.508	0.508	0.514	0.573	**1.439**	0.767
Homsap TRBV4-1 F (F)	2.597	2.027	0.242	0.319	0.319	0.359	0.418	1.300	0.629
Homsap TRBV6-4 F	2.568	1.990	0.205	0.291	0.291	0.332	0.396	1.300	0.629
Homsap TRBV11-2 F [F] (F)	2.553	1.972	0.186	0.282	0.282	0.318	0.388	1.300	0.629
Homsap TRBJ1-5 F	-2.480	1.881	0.096	0.200	0.203	0.257	0.328	1.266	0.594
Homsap TRBV12-4 F (F)	2.475	1.875	0.090	0.200	0.203	0.253	0.328	1.266	0.594
Homsap TRBV9 F (F)	2.233	1.593	0.000	0.000	0.010	0.100	0.158	1.012	0.340
Homsap TRBV12-3 F	2.035	1.378	0.000	0.000	0.010	0.033	0.069	0.823	0.151
**CD4$^-$(MID4)∼CD4$^+$(MID5) [d3]**									
Homsap TRBV5-1 F (F)	-8.032	15.000	**13.244**	**13.244**	**13.244**	**13.229**	**13.229**	**13.244**	**12.578**
Homsap TRBV27 F	7.149	12.059	**10.289**	**10.296**	**10.296**	**10.289**	**10.296**	**10.692**	**10.023**
Homsap TRBV7-2 F (F)	-7.126	11.985	**10.214**	**10.229**	**10.229**	**10.214**	**10.229**	**10.692**	**10.023**
Homsap TRBJ2-7 F ORF	5.684	7.881	**6.110**	**6.133**	**6.133**	**6.110**	**6.133**	**6.712**	**6.043**
Homsap TRBV7-9 F (F)	5.436	7.264	**5.493**	**5.523**	**5.523**	**5.493**	**5.523**	**6.192**	**5.523**
Homsap TRBV4-1 F (F)	5.372	7.108	**5.337**	**5.376**	**5.376**	**5.337**	**5.376**	**6.116**	**5.447**
Homsap TRBJ2-5 F	-5.342	7.037	**5.267**	**5.313**	**5.313**	**5.267**	**5.313**	**6.112**	**5.443**
Homsap TRBV4-3 F (F)	3.929	4.069	**2.299**	**2.353**	**2.353**	**2.300**	**2.354**	**3.202**	**2.533**
Homsap TRBJ2-1 F	3.533	3.386	**1.615**	**1.679**	**1.679**	**1.621**	**1.683**	**2.570**	**1.901**
Homsap TRBV20-1 F (F)	-3.427	3.214	**1.443**	**1.515**	**1.515**	**1.451**	**1.522**	**2.443**	**1.775**
Homsap TRBV15 F (F)	3.326	3.055	1.284	**1.365**	**1.365**	1.295	**1.374**	**2.326**	**1.657**
Homsap TRBV7-6 F (F)	3.105	2.721	0.950	1.040	1.040	0.974	1.059	**2.030**	**1.361**
Homsap TRBV12-4 F (F)	2.980	2.540	0.769	0.868	0.868	0.805	0.897	**1.883**	1.215
Homsap TRBV24-1 F	-2.823	2.323	0.552	0.660	0.660	0.610	0.706	**1.698**	1.029
Homsap TRBJ1-5 F	-2.522	1.933	0.162	0.280	0.280	0.301	0.387	**1.347**	0.678
Homsap TRBJ2-2 F	2.506	1.913	0.143	0.270	0.270	0.288	0.379	**1.347**	0.678
Homsap TRBV9 F (F)	2.428	1.819	0.048	0.185	0.185	0.226	0.317	1.279	0.610
Homsap TRBV18 F	-2.381	1.763	0	0.140	0.140	0.192	0.285	1.247	0.579
Homsap TRBV25-1 F	2.354	1.731	0	0.118	0.118	0.174	0.270	1.238	0.570
Homsap TRBJ1-3 F	-2.204	1.560	0	0	0.008	0.093	0.172	1.090	0.421
Homsap TRBV14 F (F)	2.168	1.520	0	0	0.008	0.078	0.157	1.072	0.403
Homsap TRBD2 F	-2.053	1.397	0	0	0.008	0.041	0.103	0.988	0.319
Homsap TRBD1 F	2.053	1.397	0	0	0.008	0.041	0.103	0.988	0.319
Homsap TRBV12-3 F	-1.963	1.304	0	0	0.008	0.022	0.076	0.913	0.245
**CD4$^-$(MID7)∼CD4$^+$(MID8) [d8]**									
Homsap TRBV27 F	7.400	12.866	**11.095**	**11.095**	**11.095**	**11.095**	**11.095**	**11.095**	**10.426**
Homsap TRBJ2-7 F, ORF	6.179	9.191	**7.420**	**7.427**	**7.427**	**7.420**	**7.427**	**7.847**	**7.178**
Homsap TRBV5-1 F (F)	-6.161	9.140	**7.369**	**7.384**	**7.384**	**7.369**	**7.384**	**7.847**	**7.178**
Homsap TRBV4-1 F (F)	5.406	7.191	**5.420**	**5.443**	**5.443**	**5.420**	**5.443**	**6.023**	**5.354**
Homsap TRBV7-2 F (F)	-4.929	6.082	**4.312**	**4.342**	**4.342**	**4.312**	**4.342**	**5.011**	**4.342**
Homsap TRBJ2-5 F	-4.400	4.965	**3.194**	**3.233**	**3.233**	**3.195**	**3.233**	**3.973**	**3.304**
Homsap TRBJ2-1 F	3.672	3.619	**1.848**	**1.894**	**1.894**	**1.851**	**1.897**	**2.693**	**2.024**
Homsap TRBV7-9 F (F)	3.481	3.301	**1.530**	**1.585**	**1.585**	**1.537**	**1.591**	**2.434**	**1.765**
Homsap TRBJ2-3 F	-3.176	2.825	1.054	1.118	1.118	1.073	1.134	**2.009**	**1.340**
Homsap TRBJ1-1 F	-3.073	2.674	0.903	0.975	0.975	0.930	0.998	**1.903**	1.235
Homsap TRBJ2-2 F	2.827	2.327	0.557	0.637	0.637	0.615	0.685	**1.598**	0.929

Homsap TRBV25-1 F	2.607	2.039	0.268	0.358	0.358	0.379	0.448	**1.348**	0.679
Homsap TRBV4-3 F (F)	2.547	1.965	0.194	0.292	0.292	0.324	0.397	**1.308**	0.639
Homsap TRBV5-4 F (F)	-2.451	1.847	0.076	0.184	0.193	0.244	0.316	1.252	0.583
Homsap TRBV7-7 F (F)	-2.451	1.847	0.076	0.184	0.193	0.244	0.316	1.252	0.583
Homsap TRBV9 F (F)	2.371	1.751	0.000	0.107	0.107	0.185	0.263	1.198	0.529
Homsap TRBV20-1 F (F)	-2.360	1.738	0.000	0.104	0.104	0.178	0.261	1.198	0.529
Homsap TRBV12-3 F	-2.221	1.580	0.000	0.000	0.058	0.101	0.171	1.064	0.395
Homsap TRBV18 F	-2.046	1.390	0.000	0.000	0.058	0.039	0.087	0.898	0.229
**CD4$^-$(MID10)$\sim$CD4$^+$(MID11) [d26]**									
Homsap TRBV27 F	10.631	25.669	**23.884**	**23.884**	**23.884**	**23.896**	**23.910**	**23.884**	**23.212**
Homsap TRBJ2-7 F, ORF	8.398	16.343	**14.558**	**14.565**	**14.565**	**14.556**	**14.572**	**14.859**	**14.187**
Homsap TRBV7-2 F (F)	-7.669	13.770	**11.974**	**11.989**	**11.989**	**11.973**	**11.988**	**12.451**	**11.780**
Homsap TRBV5-1 F (F)	-7.454	13.041	**11.257**	**11.279**	**11.279**	**11.257**	**11.279**	**11.859**	**11.187**
Homsap TRBV4-1 F (F)	6.332	9.618	**7.832**	**7.862**	**7.862**	**7.832**	**7.862**	**8.531**	**7.859**
Homsap TRBV4-3 F (F)	5.234	6.779	**4.994**	**5.031**	**5.031**	**4.994**	**5.031**	**5.772**	**5.100**
Homsap TRBJ2-1 F	5.114	6.502	**4.717**	**4.762**	**4.762**	**4.717**	**4.762**	**5.562**	**4.890**
Homsap TRBJ2-5 F	-5.008	6.260	**4.475**	**4.528**	**4.528**	**4.475**	**4.528**	**5.378**	**4.706**
Homsap TRBV20-1 F (F)	-4.980	6.198	**4.412**	**4.473**	**4.473**	**4.412**	**4.473**	**5.367**	**4.695**
Homsap TRBV6-2 F (P)	4.860	5.931	**4.146**	**4.215**	**4.215**	**4.146**	**4.215**	**5.165**	**4.493**
Homsap TRBV25-1 F	4.850	5.909	**4.123**	**4.201**	**4.201**	**4.123**	**4.201**	**5.165**	**4.493**
Homsap TRBJ2-3 F	-4.069	4.325	**2.540**	**2.626**	**2.626**	**2.540**	**2.627**	**3.619**	**2.947**
Homsap TRBV12-4 F (F)	3.869	3.961	**2.176**	**2.271**	**2.271**	**2.177**	**2.272**	**3.290**	**2.618**
Homsap TRBV18 F	-3.494	3.323	**1.538**	**1.642**	**1.642**	**1.544**	**1.647**	**2.684**	**2.012**
Homsap TRBJ1-4 F	-3.354	3.099	**1.314**	**1.427**	**1.427**	**1.324**	**1.435**	**2.490**	**1.818**
Homsap TRBV6-1 F	3.167	2.813	1.028	1.150	1.150	1.047	1.165	**2.232**	**1.560**
Homsap TRBV7-9 F (F)	2.967	2.522	0.737	0.869	0.875	0.775	0.897	**1.989**	**1.317**
Homsap TRBV7-6 F (F)	2.965	2.519	0.734	0.869	0.875	0.772	0.897	**1.989**	**1.317**
Homsap TRBV13 F (F)	2.713	2.177	0.391	0.543	0.543	0.475	0.602	**1.670**	0.998
Homsap TRBV12-3 F	-2.480	1.882	0.096	0.258	0.258	0.257	0.371	**1.397**	0.726
Homsap TRBV29-1F (F)	-2.427	1.818	0.032	0.205	0.205	0.216	0.331	**1.355**	0.683
Homsap TRBJ1-2 F	-2.227	1.586	0.000	0.000	0.006	0.097	0.186	1.143	0.471
Homsap TRBV11-2 F [F] (F)	2.173	1.526	0.000	0.000	0.006	0.075	0.160	1.102	0.431
Homsap TRBV6-4 F	2.103	1.451	0.000	0.000	0.006	0.051	0.127	1.060	0.388
Homsap TRBV5-4 F (F)	-2.100	1.447	0.000	0.000	0.006	0.050	0.127	1.060	0.388
Homsap TRBV9 F (F)	2.036	1.380	0.000	0.000	0.006	0.034	0.105	1.022	0.350
Homsap TRBJ1-3 F	-2.033	1.376	0.000	0.000	0.006	0.033	0.105	1.022	0.350

Unadjusted p-values (rawp) ($-\log_{10} > 1.3$) with corresponding test statistics z-scores (<-1.96 for negative differences or >1.96 for positive differences) are shown. Adjusted p-values ($-\log_{10} > 1.3$) under the given controlling procedure are highlighted in bold and correspond to significant tests (significant differences in proportions).
